# A Systems Approach to Rheumatoid Arthritis

**DOI:** 10.1371/journal.pone.0051508

**Published:** 2012-12-11

**Authors:** Sungyong You, Chul-Soo Cho, Inyoul Lee, Leroy Hood, Daehee Hwang, Wan-Uk Kim

**Affiliations:** 1 School of Interdisciplinary Bioscience and Bioengineering, POSTECH, Pohang, Korea; 2 Research Institute of Immunobiology, Catholic Research Institute of Medical Science, Seoul, Korea; 3 Department of Internal Medicine, Catholic University of Korea, Seoul, Korea; 4 Institute for Systems Biology, Seattle, Washington, United States of America; 5 Department of Chemical Engineering, POSTECH, Pohang, Korea; 6 Integrative Biosciences and Biotechnology, POSTECH, Pohang, Korea; Baylor College of Medicine, United States of America

## Abstract

Rheumatoid arthritis (RA) is a chronic autoimmune disease that primarily attacks synovial joints. Despite the advances in diagnosis and treatment of RA, novel molecular targets are still needed to improve the accuracy of diagnosis and the therapeutic outcomes. Here, we present a systems approach that can effectively 1) identify core RA-associated genes (RAGs), 2) reconstruct RA-perturbed networks, and 3) select potential targets for diagnosis and treatments of RA. By integrating multiple gene expression datasets previously reported, we first identified 983 core RAGs that show RA dominant differential expression, compared to osteoarthritis (OA), in the multiple datasets. Using the core RAGs, we then reconstructed RA-perturbed networks that delineate key RA associated cellular processes and transcriptional regulation. The networks revealed that synovial fibroblasts play major roles in defining RA-perturbed processes, anti-TNF-α therapy restored many RA-perturbed processes, and 19 transcription factors (TFs) have major contribution to deregulation of the core RAGs in the RA-perturbed networks. Finally, we selected a list of potential molecular targets that can act as metrics or modulators of the RA-perturbed networks. Therefore, these network models identify a panel of potential targets that will serve as an important resource for the discovery of therapeutic targets and diagnostic markers, as well as providing novel insights into RA pathogenesis.

## Introduction

Rheumatoid Arthritis (RA) is a chronic autoimmune disease that primarily attacks synovial joints. In the RA joints, various inflammatory cells, including innate immune cells (e.g. mast cells, macrophages, dendritic cells, and NK cells), adaptive immune cells (T- and B-cells), and fibroblast-like synoviocytes (FLS), are activated. These cells interact with each other via an array of cytokines and/or cell-to-cell contacts, leading to prolonged inflammation, abnormal proliferation of FLS, and the destruction of cartilage and bone [1], [2], [3]. Despite incremental advances in the diagnosis and treatment of RA, novel molecular targets are still needed to improve the accuracy of diagnosis and the therapeutic outcomes. For example, two metrics widely used to assess RA activity, i.e., erythrocyte sedimentation rate (ESR) and C-reactive protein (CRP), are not specific to RA because they also are elevated in non-RA conditions including infections and trauma. In addition, rheumatoid factor and anti-CCP antibody, well-known diagnostic markers for RA, represent B-cell hyperactivity to self-antigens, but are limited in reflecting the multi-cellular communication networks occurring in the RA joints.

Systems approaches to diseases postulate that diseases arise from disease-perturbed networks. Accordingly, to understand fundamental mechanisms of RA pathogenesis, it is essential to identify and analyze RA-perturbed networks in the RA synovium. Several studies have identified RA-associated genes (RAGs) and their associated cellular processes [4], [5], [6]. For example, Hurber et al. [4] analyzed mRNA expression profiles in the synovial tissues of RA patients and normal controls. They identified 568 RAGs that are mainly involved in inflammation, proliferation, survival, and angiogenesis. Van der Pouw Kraan et al. [5] and Ungethuem et al. [6] also identified RAGs participating in similar cellular processes. However, these studies have not attempted to reconstruct RA-perturbed networks that delineate cellular processes associated with RA and to identify molecular targets for diagnosis or therapy through analyses of RA-perturbed networks.

In this study, we introduce a systems approach that can be used to effectively 1) identify core RAGs by integrating multiple gene expression datasets previously reported and their associated cellular processes, 2) reconstruct RA-perturbed networks to delineate key cellular processes and transcriptional regulation associated with RA, and 3) identify targets for use in diagnosis and treatments of RA. The RA-perturbed networks revealed that 1) RA FLS act as a major player responsible for various RA-perturbed processes, 2) anti-TNF-α therapy moves a wide spectrum of RA-perturbed processes toward normality, and 3) 19 key transcription factors (TFs) could play critical roles in the regulation of 55% of dysregulation encoded by the RA-perturbed networks. Based on the RA-perturbed networks, we selected a list of potential molecular targets that can act as metrics or modulators of RA-perturbed networks. Therefore, our systems approach provides RA-perturbed network models that can identify a panel of potential targets that will serve as an important resource for discovery of therapeutic targets and diagnostic markers, as well as providing novel insights into RA pathogenesis.

**Table 1:** 14 gene expression datasets used in this study.

Sample Origin	Sample Type and Numbers	Raw DataSource ID	Reference
**Synovial Tissues**	5 Normal, 5 RA and 5 OA	GSE1919	Ungethuem et al., Physiol Genomics., 2010.
	4 Normal and 5 RA	GSE7307	none
	3 Normal, 12 RA and 9 OA	GSE12021	Huber et al., Arthritis Res Ther., 2008.
	11 RA: good, moderate, and poor responders with post-adalimumab therapy	GSE15602	Badot et al., Arthritis Res Ther., 2009.
	10 RA: good, moderate, and poor responders with pre-or post-infliximab therapy	E-TABM-104	Lindberg et al., Arthritis Res Ther., 2006.
**Peripheral T cell**	8 RA and 10 Control	GSE4588	none
**Peripheral B cell**	7 RA and 9 Control	GSE4588	none
	1 RA and 1 Control	GSE4255	Szodoray et al., Rheumatology., 2006.
**PBMC**	18 RA and 15 Control	GSE15573	Teixeira et al., PLoS One., 2009.
	27 RA and 22 Control	GSE11827	none
**FLS from synovial** **tissue**	6 RA and 6 OA	GSE7669	Pohlers et al., Arthritis Res Ther., 2007.
	19 RA and 19 Control	GSE4061	Kasperkovitz et al., Arthritis & Rheumatism., 2005.
	6 RA (1 Control, 2 IL1B Treatment, and 3 TNF Treatment)	GSE15615	Badot et al., Arthritis Res Ther., 2009.
	3 RA (1 Control, 1 IL1B Treatment, and 1 TNF Treatment)	none	Taberner et al., Inflammation Research., 2005.

## Materials and Methods

### Identification of RA Associated Genes (RAGs)

Log2-intensities in each of three gene expression datasets generated from synovial tissues (Table 1) were normalized using GC-RMA method [7], [8]. For each dataset, log2-fold-changes for each probe set in *m* RA samples were then calculated by subtracting the median intensity of normal synovial tissue samples from *m* intensities of RA samples, resulting in a *k*×*m* fold-change matrix where *k* is the number of probe sets. The same procedure is done separately for OA samples, resulting in another *k*×*n* fold difference matrix. Then, for each fold-change matrix, when there are multiple probe sets for a gene, the representative fold-change for the gene was selected as that of the probe set with the largest log2-fold-change between RA (or OA) and normal samples.

**Figure 1:**
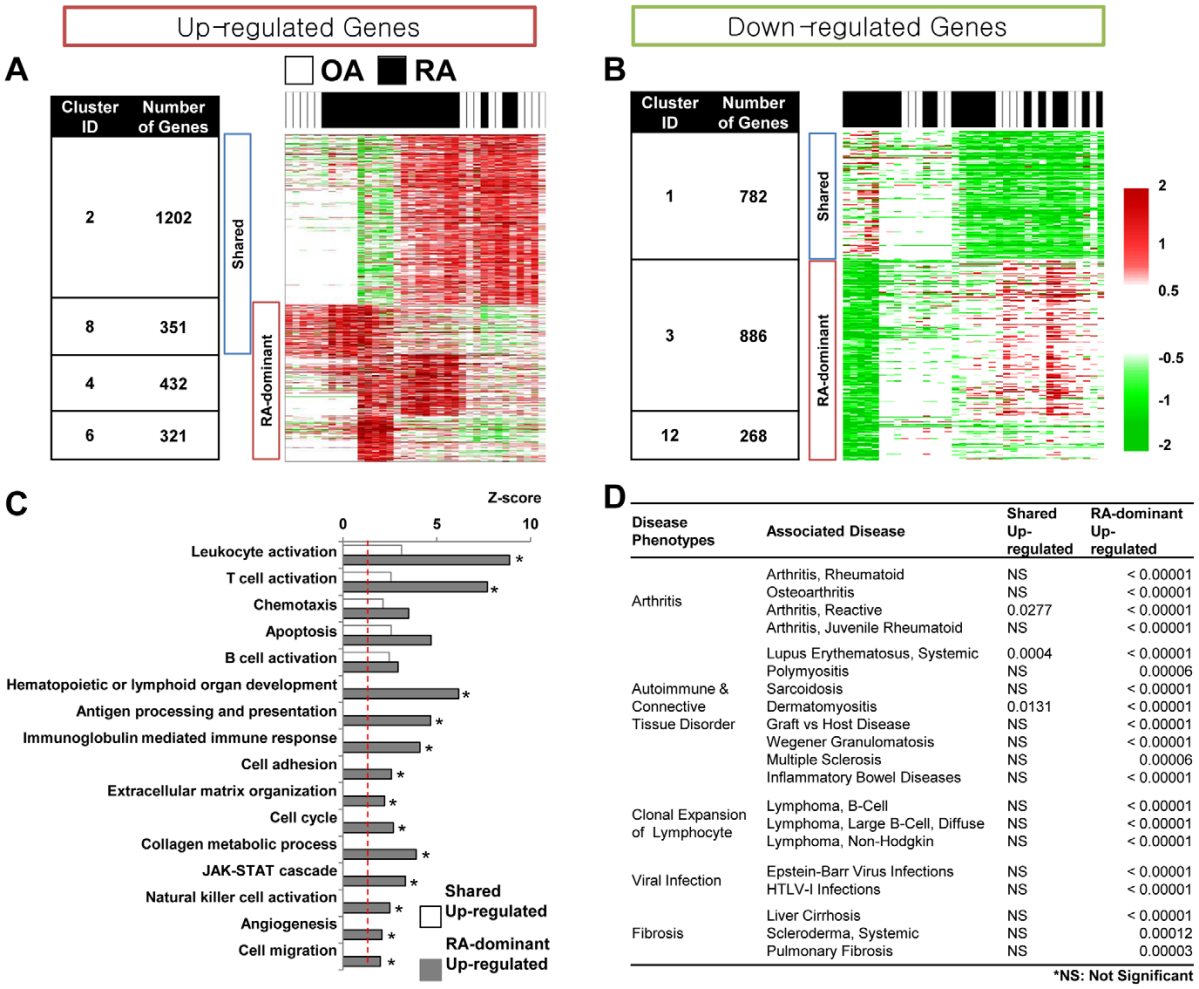
RA associated genes, cellular processes and disease phenotypes. A) and B) Seven major clusters (1, 2, 3, 4, 6, 8, 12) showing the DEPs of the RAGs in RA and OA samples: Shared (shared RAGs commonly up- or down-regulated in RA and OA samples; RA-dominant (RAGs dominantly up- or down-regulated in RA samples). The number of RAGs in each cluster is denoted in the table. When a gene shows a mixture of the DEPs in the multiple clusters, NMF, as a soft clustering method, assigns the gene to multiple clusters. Thus, the summation of the RA-dominant up-regulated RAGs (1104 RAGs) could be larger than 983 presented as the number of the RA-dominant RAGs. C) GO Biological Processes (GOBPs) enriched by the up-regulated RAGs (*P*<0.05). For each GOBP, a Z score was computed by *N* ^−1^(1-*P*) where *N* ^−1^(−) is the inverse of a standard normal cumulative density function and *P* is the enrichment p-value for the GOBP. Empty and gray bars represent the GOBPs enriched by shared and RA-dominant up-regulated RAGs, respectively. D) Five classes of RA-related diseases and their association with the RAGs. P-values were computed using the empirical statistical testing described in supplementary methods).

In total, the five fold-change matrices were generated (i.e., two from GSE1919, one from GSE7307, and two from GSE12021). Each of these five matrices was then transformed into a single vector. The five vectors for the five matrices were normalized using quantile normalization method [8] to avoid a bias toward certain datasets with large fold changes. These vectors with normalized fold-changes were transformed back into the matrices. Finally, the five normalized fold-change matrices were combined by matching the gene IDs in the individual matrices, resulting in a combined fold-change matrix. NMF was then applied to the combined fold-change matrix as described in Kim et al. [9]. The number of clusters (*k*) was set to be 30. Figure S1 shows the 30 resulting clusters showing the corresponding DEPs. The genes belonging to cluster *i* (i.e. the gene showing the DEP in cluster *i*) were selected as the ones whose adjusted P-values values are less than 0.05. We computed the adjusted P-values as described in Kim et al. [9].

**Figure 2:**
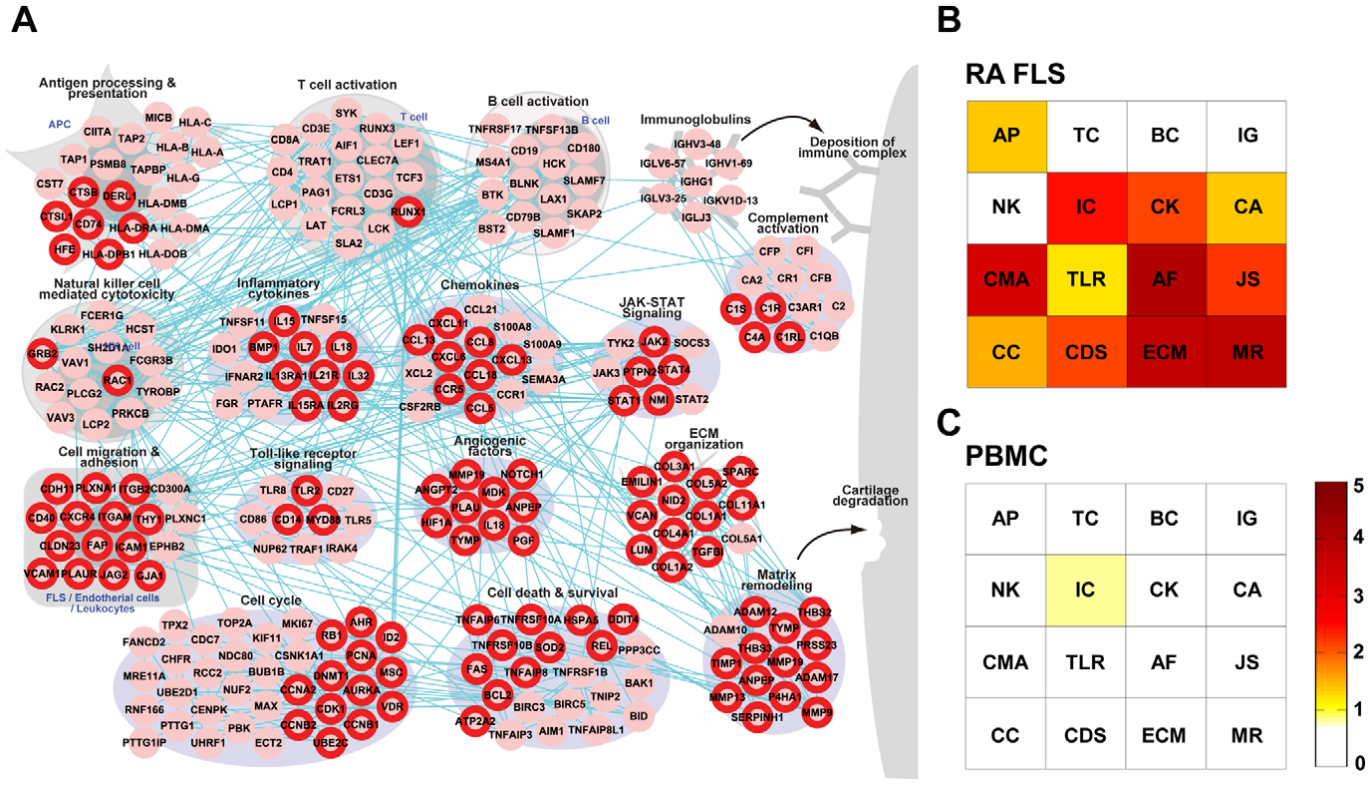
A RA-perturbed network in the RA synovium and signatures of FLS and PBMC in the RA tissue network. A) A RA-perturbed network describing RA associated cellular processes in which 242 up-regulated RAGs are involved and their interactions. The network nodes are arranged into sixteen modules based on their GOBPs and the KEGG pathways that they belong to. The nodes with red boundary represent DEGs in RA FLS. B) and C) Module enrichment scores (see text for definition) representing the significances of overlaps of the DEGs in RA FLS (B) or PBMC (C) with the genes belonging to the sixteen network modules. See text for detailed discussion. AP = Antigen processing & presentation; TC = T-cell activation; BC = B-cell activation; IG = Immunoglobulins; CA = Complement activation; NK = Natural killer cell mediated cytotoxicity; IC = Inflammatory cytokines; CK = Chemokines; CMH = Cell migration & adhesion; TLR = Toll-like receptor signaling; AF = Angiogenic factors; JS = JAK-STAT signaling; CC = Cell cycle & DNA repair; CDS = Cell death & survival; ECM = ECM organization; MR = Matrix remodeling.

### Selection of the Major Differential Expression Patterns (DEPs)

We selected seven significant DEPs that are not likely to be observed by chance using the following procedure. We first randomly permuted the elements of the log2-fold-change matrix and then applied NMF clustering to the randomly permuted matrix with the number of clusters (*k*) = 30. This permutation experiment results in random expression patterns, compared to the 30 RA-associated DEPs in Figure S1. In each random permutation experiment, we computed the cutoffs of both NMF activation and basis values as 95^th^ percentile values and then counted genes and samples with the basis and activation values larger than the cutoffs. We found that 211 genes in five samples can show a DEP by chance (P<0.05). Thus, we selected seven DEPs in which 1) the number of genes in a certain DEP should be more than 211, and 2) the number of samples with the DEP is more than five, and then identified 3742 RAGs showing these DEPs.

**Figure 3:**
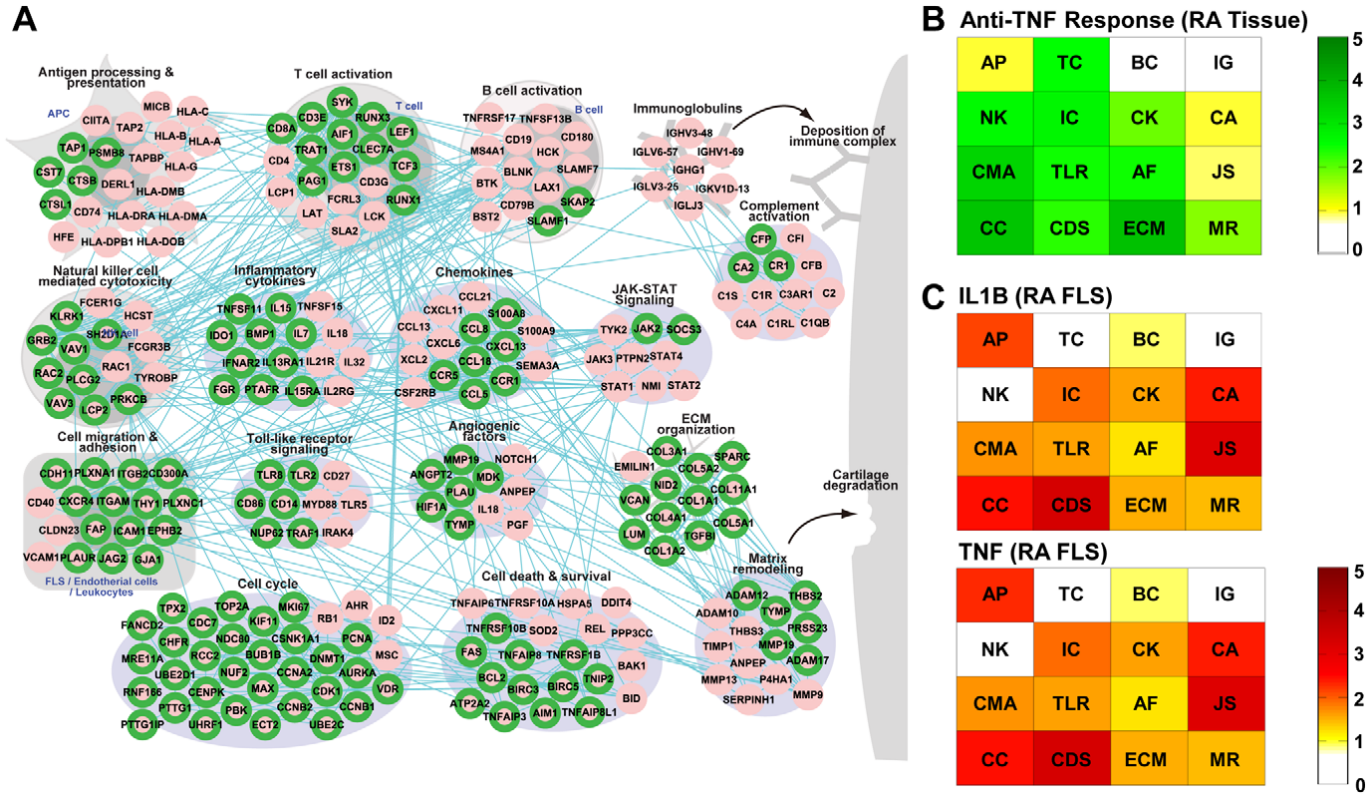
Signatures of anti-TNF inhibitors in RA-perturbed network. A) A RA-perturbed networks showing the recovery of the elevated RAGs to normality by anti-TNF therapy. Green border colors represent the decreases in expression levels of 136 elevated RAGs. B) and C) Module enrichment scores representing the significances of overlaps of the genes decreased by anti-TNF therapy (B) or the genes whose expression levels are elevated by IL1B and TNF treatments (C) with the genes belonging to the network modules. AP = Antigen processing & presentation; TC = T-cell activation; BC = B-cell activation; IG = Immunoglobulins; CA = Complement activation; NK = Natural killer cell mediated cytotoxicity; IC = Inflammatory cytokines; CK = Chemokines; CMH = Cell migration & adhesion; TLR = Toll-like receptor signaling; AF = Angiogenic factors; JS = JAK-STAT signaling; CC = Cell cycle & DNA repair; CDS = Cell death & survival; ECM = ECM organization; MR = Matrix remodeling.

### Identification of Diseases and their Phenotypes Associated with RAGs

We collected gene and disease/disease phenotype association data from the Gendoo database [10]. For 1539 shared up-regulated RAGs, we then counted the number of genes associated with each disease and disease phenotype (i.e. *n* genes had disease phenotype *i*, such as Arthritis and Rheumatoid, according to the association information). Second, we randomly sampled 1539 genes from the whole genome and then counted randomly sampled genes with phenotype *i*. We repeated this procedure 100,000 times. Third, we then generated an empirical distribution (null hypothesis distribution) of the 100,000 counts of the randomly sampled genes with phenotype *i*. Fourth, for each association (e.g. *n* gene-phenotype *i*), we then computed the probability (P) that the actual count of genes with phenotype *i* can be observed by chance using one-tailed test with the empirical distribution. The same procedure was repeated for all the pairs of gene-disease/disease phenotype found for the 1539 RAGs. Finally, we selected a list of disease/disease phenotype associations enriched by the 1539 RAGs as the ones P<0.05. The same procedure was done for the 983 RA-dominant up-regulated RAGs.

**Figure 4:**
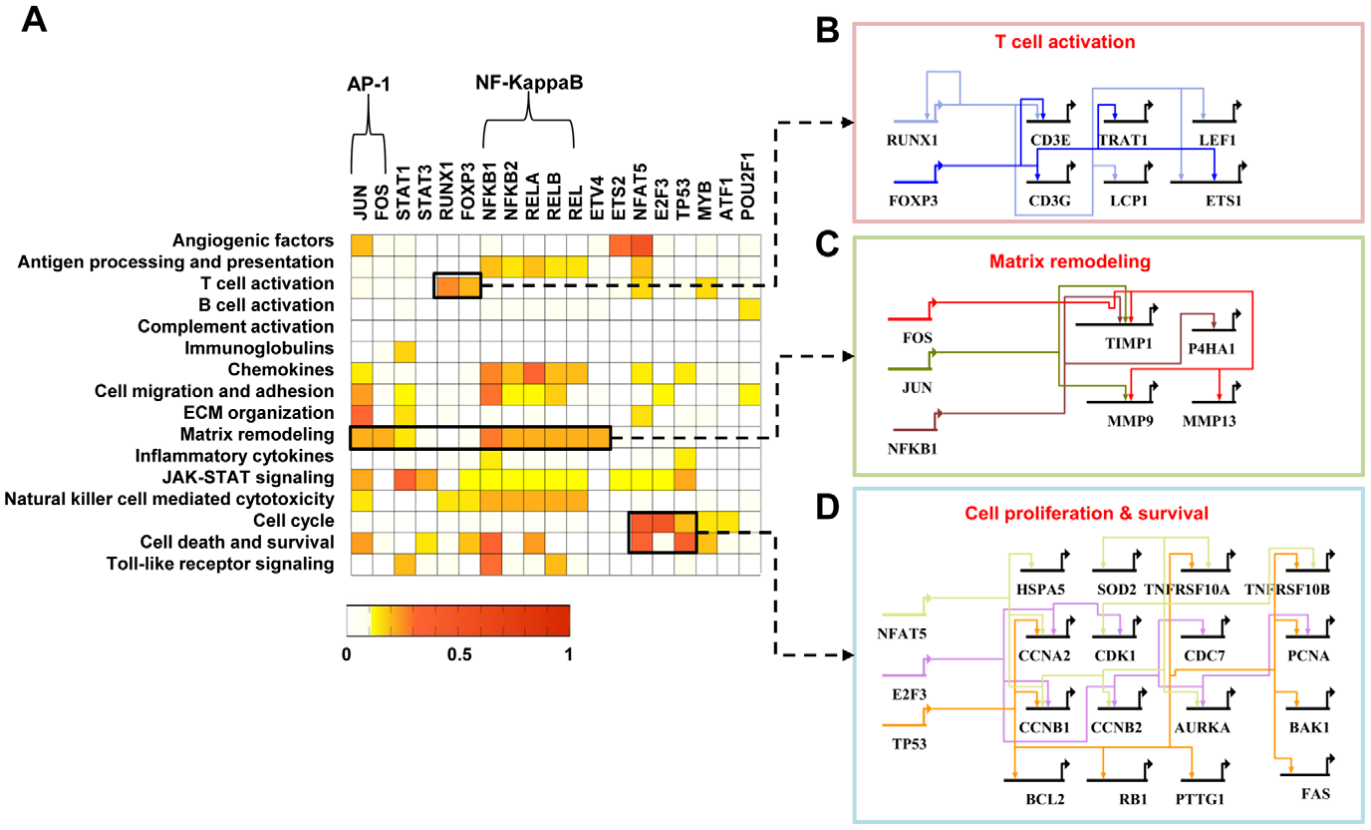
Gene regulatory networks activated in RA. A) Target enrichment scores representing the significances of overlaps between the targets of each TF and the RAGs belonging to the network modules. B–D) Gene regulatory networks describing the TF-target relationships for the three processes: T-cell activation including RUNX1 and FOXP3 (B), Matrix remodeling including AP-1 (JUN and FOS) and NFKB1 (C), and Cell proliferation and survival including NFAT5, E2F3, and TP53 (D).

### Reconstruction of RA-perturbed Networks

To reconstruct an RA-perturbed network, from the 983 RA-dominant up-regulated RAGs, we first selected 242 RAGs which are composed of 131 RAGs that are involved in sixteen RA associated cellular processes and their 111 interactors based on the interaction data obtained from public databases including HPRD [11], BioGRID [12], STRING [13], and KEGG [14]. A RA-perturbed network model was then reconstructed using the interactions among the 242 RAGs. The nodes in the network were arranged such that the nodes with the same GOBPs [15] and KEGG pathways were grouped into the same network modules, resulting in the sixteen modules.

**Table 2:** Known molecular target candidates for diagnosis and therapy of RA.

Category	Symbol	P Value	Chemical	RA association	Associated Modules
PPI	PTPRC	<0.00001	O	O	T cell activation
	JAK1	<0.00001	O	O	JAK-STAT signaling
	CD19	<0.00001	O	O	B cell activation
	TNF	<0.00001	O	O	Inflammatory cytokines
	CXCR4	<0.00001	O	O	Cell migration and adhesion, Chemokines
	TLR4	<0.00001	O	O	Toll-like receptor signaling
	CD247	<0.00001	O	O	T cell activation, NK cell mediated cytotoxicity
	CCR3	0.00001	O	O	Chemokines
	THBS1	0.00027	O	O	Matrix remodeling
TF	NFKB1	<0.00001	O	O	Matrix remodeling, NK cell mediated cytotoxicity, Cell death and survival, Chemokines
	TP53	<0.00001	O	O	Cell death and survival
	JUN	0.00001	O	O	Matrix remodeling, ECM organization
	FOXP3	0.00009	O	O	T cell activation
	POU2F1	0.00028	O	O	B cell activation
	ETS2	0.00377	O	O	Angiogenic factors, Chemokines

**Table 3:** Novel molecular target candidates for diagnosis and therapy of RA.

Category	Symbol	P Value	Chemical	Associated Diseases	Associated Modules
PPI	CSF3R	<0.00001	–	Multiple Sclerosis	Inflammatory cytokines
	DOK1	<0.00001	–	Epstein-Barr Virus Infections	Cell death and survival
	KHDRBS1	<0.00001	O	HTLV-I Infections	Cell cycle
	AXL	<0.00001	O	–	Angiogenic factors
	PDGFB	<0.00001	O	Liver Cirrhosis, Non-Hodgkin Lymphoma, Multiple Sclerosis, Systemic Scleroderma	Chemokines
	RELN	<0.00001	O	Liver Cirrhosis	ECM organization
	PTK2B	0.00001	O	Lymphoma, B-Cell; Pulmonary Fibrosis	NK cell mediated cytotoxicity
TF	NFAT5	<0.00001	–	–	Cell cycle, Cell death and survival, Angiogenic factors
	E2F3	<0.00001	O	Lymphoma, Large B-Cell, Diffuse	Cell cycle

### Computation of Module Enrichment Scores

To quantitatively assess the contribution of cell types to RA pathogenesis, we integrated gene expression datasets collected from multiple types of cells related to RA pathogenesis into the RA-perturbed network. We first identified up-regulated genes (Cell Genes) in these cells and then examined how closely the up-regulated genes in these cells overlap with the RA-dominant up-regulated genes associated with the individual modules in the RA-perturbed network. For each network module, we calculated a module enrichment score (MES) defined by [(the number of Cell Genes within a module)/(the total number of genes within the module)]/[(the total number of genes in RA-perturbed network)/(the total number of RA-dominant up-regulated RAGs)]. A high MES indicates a large overlap with the genes in the corresponding network module. We also computed the MES to assess the effect of TNF-α inhibitors on restoration of RA-perturbed networks toward normality and also the effects of IL1B and TNF on induction of RA pathogenic conditions. In these cases, we used the same equation, but the numbers of down-regulated genes by TNF-α inhibitors and up-regulated genes by IL1B or TNF were used instead of the number of up-regulated genes in each type of cells.

### Identification of Key Transcription Factors (TFs)

To identify key TFs, we first collected 60,948 TF-target interaction data for 259 TFs in the public databases including TRED [16], EdgeExpressDB [17], Amadeus [18], bZIPDB [19], and OregAnno [20]. A random sampling based empirical statistical testing was applied to identify TFs significantly enriched by the 983 RA-dominant up-regulated RAGs. For each TF, we counted its targets in the 983 RAGs (e.g. *n* targets of TF *i*). Second, we randomly sampled 983 genes from the whole genome and then counted targets of TF *i* in the randomly sampled 983 genes. We repeated this procedure 100,000 times. Third, we then generated an empirical distribution (null hypothesis distribution) of the 100,000 counts of random targets of TF*i*. Fourth, for the number of targets of TF *i*, we then computed the probability (P) that the actual count of targets of TF *i* in the 983 RAGs can be observed by chance using one-tailed test with the empirical distribution. The same procedure was repeated for all TFs. Finally, we selected 19 TFs whose targets were significantly enriched by the 983 RAGs (P<0.01).

### Association of Key TFs with Network Modules

To quantitatively assess the significance of the 19 key TFs regulating cellular processes represented by individual modules in the network, we computed the target enrichment scores representing how significantly each module can be regulated by the key TFs using the previously reported TF targets. In each module, for a key TF, the target enrichment score was defined by (the number of targets regulated by the TF within a module)/(the total number of genes within a module). A high enrichment score in individual modules for a key TF indicates that a large portion of molecules in the corresponding modules are regulated by the TF.

### Identification of an Initial Set of Potential Molecular Targets

We first collected protein-protein interaction data from public databases including HPRD [11], BioGRID [12], STRING [13], and KEGG [14]. To identify a list of molecular targets that play major contribution to regulation of the RA-dominant up-regulated 983 RAGs, we used a random sampling based empirical statistical testing similar to the method described in the previous section. Specifically, 1) for each regulator, the first and second neighbors using the protein-protein interaction data was counted instead of the number of targets of TF *i*; 2) for the regulator, by randomly sampling the 983 genes 100,000 times, we generated an empirical distribution of the null hypothesis that this regulator has an insignificant number of interactions with the RAGs; 3) for the protein regulator, we used a one-tailed test on the empirical null hypothesis distribution to computed P values for the observed number of first and second neighbors; and 4) after repeating procedures 1 to 3, we selected 108 key regulators with P-values of the first and second neighbors with P<0.01 (Table S1).

## Results and Discussion

### RA Associated Genes Show Gene Expression Patterns Unique to RA

To identify RAGs, we first collected three gene expression datasets generated from synovial tissues of RA patients, osteoarthritis (OA) patients, and healthy subjects (GSE1919, GSE7307, and GSE12021 in Table 1). These datasets include a total of 12 normal, 22 RA, and 14 OA samples for 14907 genes. We then identified RAGs showing differential expression in RA samples by collectively analyzing the 48 samples in the three datasets using a non-negative matrix factorization (NMF) analysis (see **Materials and Methods**). Among 30 different differential expression pattern (DEPs) clusters of the genes resulted from the NMF analysis, we selected seven major DEP clusters (P<0.05; see **Materials and Methods**) and then identified the genes significantly showing each DEP (P<0.05) as RAGs (Figures 1A and 1B).

The RAGs could be divided into four groups (Figures 1A and 1B): 1) shared RAGs (clusters 2 and 8) up-regulated in both RA and OA samples; 2) RA-dominant RAGs (clusters 4, 6, and 8) up-regulated predominantly in RA samples; 3) shared RAGs (cluster 1) down-regulated in both RA and OA samples; and 4) RA-dominant RAGs (clusters 3 and 12) down-regulated predominantly in RA samples. We included cluster 8 in both up-regulated groups because it showed both shared and RA-dominant DEPs. The shared RAGs indicate that both RA and OA share certain pathological processes, consistent with previous findings that both diseases show common characteristics related to chronic inflammatory arthritis. For example, angiocentric infiltrates of CD3 (+) T-cells are distributed in similar patterns in the RA and OA synoviums [21]. The RA-dominant RAGs indicate that RA can be distinguished from OA. Hence, to understand the networks unique to, or at least dominant in RA, we should focus on the pathological processes associated with RA-dominant RAGs.

### RA-dominant RAGs Represent Cellular Events in RA and RA Related Diseases

RA-dominant RAGs define cellular processes predominantly perturbed in RA. To identify these cellular processes, we performed functional enrichment analyses of the four groups of RAGs above using DAVID software [22] and then compared the results. Cellular processes enriched by the two groups of up-regulated RAGs (*P*<0.05) showed that shared up-regulated RAGs in RA and OA were mainly involved in innate and adaptive immune-related processes (leukocyte activation, chemotaxis, T/B-cell activation, and apoptosis; Figure 1C). These processes were also enriched by RA-dominant up-regulated RAGs. However, the enrichment scores (Z-scores) of these processes tended to be higher than those for the shared up-regulated RAGs (e.g. leukocyte activation [23] and T-cell activation [24]). This result implies that these processes, although commonly perturbed by RA and OA, may become further intensified in RA by the RA-dominant RAGs, consistent with the previous finding that several cytochemokines and growth factors are detected in both RA and OA synoviums, but their expression levels are higher in RA synoviums [25]. Cellular processes enriched by the two groups of down-regulated RAGs (*P*<0.05) revealed that shared down-regulated RAGs in RA and OA were mainly involved in RNA processing, regulation of cell death, angiogenesis, and regulation of cell migration (Figure S2A). Both cell adhesion and insulin receptor signaling pathways were enriched by both shared and RA-dominant down-regulated RAGs. On the other hand, RA-dominant down-regulated RAGs were specifically involved in lipid metabolic process and regulation of cell growth.

Furthermore, we investigated diseases enriched by the four groups of the RAGs using the gene-disease association data obtained from Gendoo database [10]. Various diseases exhibited close association with the RAGs (*P*<0.05; see **Materials and Methods**). Five classes of diseases involving some of the cellular processes were enriched by the shared and RA-dominant up-regulated RAGs (Figure 1D); 1) arthritis diseases (juvenile chronic arthritis and reactive arthritis, in addition to RA and OA), 2) autoimmune and connective tissue disorders, including lupus and Sjögren’s syndrome, 3) diseases employing clonal expansion of lymphocytes (e.g. diffuse large cell lymphoma), 4) viral infectious diseases including EBV and HTLV-1 infection, and 5) fibrotic diseases including systemic sclerosis. Similar to the data on cellular processes, these five classes of diseases were strongly enriched by the RA-dominant RAGs, but only partially and weakly by the shared RAGs (Figure 1D). However, the analysis of the down-regulated RAGs (Figure S2B) showed that none of these RA-related diseases was enriched by the down-regulated RAGs. This indicates that association of the down-regulated RAGs with these diseases has not been well-studied yet or they could contribute to pathogenesis of the diseases less than the up-regulated RAGs.

The pathology of RA is characterized by synoviocyte proliferation and angiogenesis, ‘pannus formation’, as well as cartilage and bone destruction by activated cells [26], [27]. The analysis of disease association showed that the up-regulated RAGs were more closely associated with RA pathology than the down-regulated RAGs (Figure 1D and Figure S2B). Among the up-regulated RAGs, the functional enrichment analysis further indicated that the processes enriched by the RA-dominant up-regulated RAGs account better for RA pathology than those by the shared up-regulated RAGs. For example, the pannus formation related processes, such as cell cycle (synoviocyte proliferation) and angiogenesis, were specifically enriched in the RA-dominant up-regulated RAGs (Figure 1C). Also, processes related to cartilage and bone destruction, such as extracellular matrix (ECM) remodeling and cell migration, were significantly enriched by the RA-dominant up-regulated RAGs, but not by the shared RAGs (Figure 1C). Hence, we defined the 983 RA-dominant up-regulated RAGs as core RAGs and focused on them to effectively delineate RA-perturbed networks.

### A RA-perturbed Network Reveals Key Cell Players in RA Synovium

Using the core RAGs, we reconstructed an RA-perturbed network describing RA-associated cellular processes and their interactions (Figure 2A; see **Materials and Methods**). The nodes in the network were grouped into the sixteen modules. These modules include the innate and adaptive immune response related modules, the inflammatory cytokine and chemokine related modules, the cell proliferation and survival related modules, the immune complex deposition related modules, and the joint destruction related modules. These modules collectively explain much of the pathophysiology of RA.

The different types of immune cells related to RA pathology interact in a complex manner. This complexity presents challenges in determining the specific roles of various types of cells in the progression of RA. Elucidation of the major and minor roles of the participating cells is a key question in understanding RA pathogenesis. RA FLS have been considered as sentinel cells, albeit without direct evidence, which actively participate in joint destruction in RA [28], [29], [30], [31]. Therefore, we determined how many modules reflect the gene signatures of RA FLS. We first identified 111 up-regulated genes in RA FLS, compared to controls (i.e. OA FLS or reference mRNA samples; see Table 1), as described in Lee et al. [32]. We then denoted these genes in the RA-perturbed network. They overlapped with 46% of the 242 RAGs in the network, indicating that the RA FLS signature overlapped significantly with the RA tissue signature. To quantitatively assess the contribution of RA FLS to the RA-perturbed network, we calculated a module enrichment score (MES; see **Materials and Methods**). Many modules in the RA tissue network had high MES values, except for the modules of T/B-cell activation, immunoglobulin, and NK cell mediated cytotoxicity (Figure 2B), indicating that the FLS signature strongly contributes to the tissue pathology. In fact, this is the first evidence in human samples supporting the previous idea that FLS play central roles as a major component of invasive pannus in many molecular events occurring in the RA joints.

Peripheral blood mononuclear cells (PBMCs) can also contribute to RA progression when recruited to and activated in the joints. To investigate whether PBMCs, like RA FLS, also reflect pathological signatures in RA joints, we integrated five gene expression datasets obtained from peripheral T, B, and mononuclear cells (Table 1 and Figure S3A). We identified the up-regulated genes in each PBMC dataset, compared to those of healthy individuals, and then combined all up-regulated genes in the five datasets. We denoted these genes in the RA-perturbed network and then recomputed the MES values for the individual modules. Interestingly, the PBMCs signature showed no overlap with the RA tissue signature as indicated by the low MES values (Figure 2C), consistent with a previous finding that there was no correlation between the fold-changes of the genes in PBMCs and inflammatory status of synovial tissues in RA joints [33]. Meanwhile, the up-regulated genes in PBMCs were involved in cell proliferation and immune response related processes (Figure S3B). Although these processes as a whole were shared between RA synovial tissues and PBMCs, the genes themselves were not shared. These results demonstrated that the PBMCs signatures show little reflection of joint pathology, a finding that might be explained by the exposure of PBMCs to a micro-environment different from that of the joints.

### The RA-perturbed Network is Restored by TNF-α Inhibitors

Biologic agents, including TNF-α inhibitors and B-cell ablating agents (e.g. anti-CD 20 antibody), have been widely used for the treatment of RA. An interesting question is whether the RA-perturbed networks can serve as a basis for understanding of the therapeutic effects of these drugs. To answer this question, we first identified a set of genes that are down-regulated by TNF-α inhibitors in synovial tissues of RA patients after treatments with anti-TNF-α antibodies (Table 1) and then denoted these genes in the RA-perturbed network. The result showed that 136 (56%) of the 242 up-regulated RAGs in the network were significantly decreased (P<0.05) in their expression levels by anti-TNF-α therapy (Figure 3A). Moreover, the majority of anti-TNF-α-regulated modules had high MES values (Figure 3B), supporting why TNF-α inhibitors are effective for most RA patients. As expected, innate immunity-related modules, including natural killer cell mediated cytotoxicity and the inflammatory cytokines module, were significantly affected by anti-TNF-α therapy (Figure 3B). Of note, the ‘pannus’-related modules, including cell migration and adhesion, cell cycle, and ECM organization, were most significantly reduced by anti-TNF-α antibody treatment, implying that the elevated TNF-α may be necessary for the formation of invasive pannus. However, B-cell-related modules, such as the B-cell activation and the immunoglobulin modules, were only modestly changed by anti-TNF-α therapy (Figure 3B), suggesting that B-cell-targeted therapy may be effective for the anti-TNF-α resistant cases. Indeed, rituximab, anti-CD20 monoclonal antibody, has been approved for the treatment of RA patients who are refractory to TNF-α inhibitors [34], [35], [36].

The heterogeneous responses of RA patients to anti-TNF-α therapy raise the possibility that other cytokines such as IL-1β may dominate joint inflammation over TNF-α in certain circumstances. We thus analyzed the up-regulated genes in TNF-α or IL-1β-stimulated RA FLS, compared to un-stimulated RA FLS (Table 1). We then integrated these genes into the RA-perturbed network. The effect of TNF-α in the RA-perturbed network is very similar to that of IL-1β (Figure 3C), implying that IL-1β and TNF-α appear to play similar pathological roles in RA. Thus, it is not surprising that anakinra, an IL-1 receptor antagonist, shows no therapeutic benefit in RA patients resistant to TNF blockades [37]. Taken together, our data suggest that molecular signatures in the RA synovium could provide important metrics to decide which kinds of biologic agents should be administered to diverse subgroups of RA patients.

### A Transcriptional Regulatory Network Reveals Key TFs Governing Regulation of RA-dominant RAGs

To elucidate key TFs that control many 983 RA-dominant RAGs and thus presumably regulate RA, we also reconstructed transcriptional regulatory networks (TRNs). We first identified 19 key TFs governing regulation of the 983 RA-dominant RAGs using previously reported TF-target interaction data (*P*<0.01; see **Materials and Methods**). The targets of 19 key TFs accounted for 55% of the 242 RAGs in the RA-perturbed network.

Using the TF-target interaction data previously reported, we then counted the numbers of targets of key TFs in the individual network modules (Figure 4A) to understand how significantly the TFs regulate the cellular functions represented by the network modules (see **Materials and Methods**). First, FOXP3 and RUNX1 act as major regulators of T-cell activation (Figure 4B), directing the expression of CD3E, CD3G, TRAT1, LCP1, LEF1, and/or ETS1. FOXP3 was shown to regulate key genes during T-cell stimulation and maturation [38], [39]. Especially, regulatory T cells expressing FOXP3 play critical roles in regulation of immune-mediated inflammation and autoimmune disorders [40]. RUNX1 was also known to modulate the differentiation of naive CD4-positive T cells [41], [42]. Second, both AP-1 (JUN and FOS) and NF-κB complexes (NF-κB1, NF-κB2, RELA, RELB, and REL) were found to regulate most significantly several network modules, including angiogenic factors, matrix remodeling, cell death and survival, and chemokines module (Figure 4A), as previously reported [43]. For example, AP-1 and NF-κB collectively regulate the genes involved in matrix remodeling (Figure 4C), including the MMP-9, MMP13, TIMP1, and P4HA1. JUN and FOS are highly expressed in RA synovial tissues [44]. Moreover, the AP-1 decoy oligonucleotides suppressed collagen-induced arthritis and inhibited production of IL-1, IL-6, TNF-α, matrix metalloproteinase (MMP)-3, and MMP-9 in RA synovial tissues [45]. NF-κB is also activated in the RA synovium and induces a battery of inflammatory genes, including TNF-α, IL-6, IL-8, and inducible nitric oxide synthase (iNOS) [43], [46]. Also, NF-κB inhibitors relieved arthritis symptoms and prevented the radiographic progression of RA patients [47], [48]. Third, NFAT5, E2F3, and TP53 can regulate the genes associated with cell cycle, cell death, and survival (Figure 4D), including CCNB1/2, CDK1, RB1, PCNA, PTTG1, BCL2, FAS, and TNFRSF10A. Mutations of TP53 tumor suppressor have been frequently noted in RA synovial tissues and synoviocytes [49], [50], [51]. Micro-dissection of RA synovium can localize islands of TP53 mutant cells to the intimal lining that exhibit higher expression of IL-6 than wild-type regions [52]. These data indicate that the 19 key TFs could be activated in RA, and the TRNs highlight further regulation of their target RAGs and cellular processes. In addition, the transcriptional regulation of the target genes in the network modules (Figure 4A) may be useful when we attempt to design drugs that can control specific modules in the RA-perturbed network.

### Potential Molecular Targets that can Modulate Activities of RA-perturbed Networks

Based on the RA-perturbed networks (Figures 2 and 4), we sought to identify candidates for molecular targets that can be used for diagnosis and therapy. They should be metrics (and modulators) of the RA-perturbed network activities. In this search, we hypothesized that a network node with a larger number of protein-protein and/or protein-DNA interactions could serve more effectively as a metric of network activation, and that its perturbation may more significantly modulate the activities of the RAGs and the RA-perturbed networks. After counting the number of interactions for every up-regulated RAG, we identified an initial set of 108 candidates with the numbers of interactions being significantly higher than those observed by chance from 100,000 random sampling experiments (*P*<0.01; see **Materials and Methods**).

Among these 108 candidates (Table S1) and the 19 TFs selected above (Table S2), we further selected two sets of molecular candidates. The first set of 15 known candidates were identified as the ones (Table 2); 1) that are currently being used as therapeutic targets in RA treatments or whose efficacy has been previously reported in RA, and 2) for which the agents modulating their activities are available. If there were multiple candidates in the same module, the candidate with the smallest *P* value was chosen with a high priority. The list includes TNF-α whose inhibition is highly efficacious, as well as CXCR4, PTPRC, and CD19 that have been previously proposed as promising drug targets [53], [54], [55]. Interestingly, PTPRC mutations have been reported to be associated with responses to anti-TNF-α therapy in RA [55]. Although the listed candidates are known as potential therapeutic targets, most of their inhibitors have neither been tested nor proven effective by a clinical study. Our network analysis further indicates that the inhibitors of these targets could be tested singly or in combination with other drugs. We further identified the second set of candidates (Table 3) that have never been reported as diagnostic markers or therapeutic targets of RA though they represent RA-associated cellular processes (Figure 1C). We showed above that ‘pannus formation’ related processes (cell cycle, cell migration and adhesion, angiogenesis, and ECM organization) were specifically enriched by RA-dominant RAGs (Figure 1C). Thus, we selected nine candidates representing these processes. The candidates have been implicated in RA-related diseases, such as multiple sclerosis and lymphoma, (Figure 1D), but their roles in RA have never been reported and thus should be confirmed *in vitro and in vivo*.

Among the candidates, we have previously reported experimental testing on the role of NFAT5, known as an osmoprotective TF activated by hypertonicity, in RA pathogenesis using human RA FLS and also in heterozygous NFAT5^+/−^ mice [56]. The results showed that NFAT5 was highly expressed in RA synoviums, and its activity was increased by proinflammatory cytokines. Further, we found that the heterozygous NFAT5^+/−^ mice exhibited a nearly complete suppression of experimentally-induced arthritis. Gene expression profiling and *in vitro* assays also revealed that NFAT5 knock-down RA FLS and endothelial cells showed the significant decreases in proliferation/survival and cell migration, respectively. This example demonstrates that the candidates in Table 2B may offer new options for diagnosis and the treatment of RA.

Therapeutic options are limited for the RA patients who are refractory to biologics and combinatory treatment with disease-modifying anti-rheumatic drugs (DMARDs). Moreover, conventional DMARDs must be discontinued within one year for many RA patients because of drug toxicity or therapy-independent relapse [57]. Thus, we require new target molecules for the treatment of RA. We expect that the above molecular candidates may offer new therapeutic options through 1) suppression of unrecognized critical pathways involved in RA, 2) additional inhibition of known pathologic pathways when used together with current drugs, and/or 3) prevention of the resistance pathway to the previous drugs. In addition, as potential diagnostic markers, these candidates can provide fundamental information on the disease state. Furthermore, some of these molecules that are secreted into blood could serve as serum diagnostic markers. Therefore, these candidates are worthy of further investigation on a large scale in that they may overcome some of the current limitations to diagnosis and treatment of RA.

### Conclusion

Several molecules have been used for diagnosis and treatment of RA. However, novel molecular targets are still needed to improve the accuracy of diagnosis and the therapeutic outcomes. In this study, we introduced a systems approach for the identification a panel of potential targets that can be used for diagnosis and treatment of RA. This approach first provided a comprehensive list of potential molecular targets as RA-dominant RAGs associated with the activation of immune-related processes and ‘pannus formation’ related processes. The approach further provided the RA-perturbed networks showing the relationships among the RA-dominant RAGs. These networks shed novel insights into RA pathogenesis; in this study, we showed that RA FLS act as a major player in ‘pannus formation’, and that anti-TNF-α therapy moves many RA-perturbed processes toward normality. Finally, among the RA-dominant RAGs, the approach provided a panel of potential molecules selected by analyzing the RA-perturbed networks, which could serves as an important resource for discovery of therapeutic targets and diagnostic markers. We expect that this approach should be applicable to other complex autoimmune diseases, such as autoimmune hepatitis and lupus nephritis, for which the core networks are not known and for which new options for diagnosis and therapy are needed. In conclusion, our approach offers new opportunities for enhancing our understanding of complex diseases and also provides a panel of molecular targets that significantly affect activities of disease-perturbed networks.

## Supporting Information

Figure S1 **NMF clustering results.**
(TIF)

Figure S2 **Functional enrichment analysis and disease association analysis for the down-regulated RAGs.** A) GOBPs enriched by the shared and RA-dominant down-regulated RAGs (*P*<0.05). B) Association of five classes of RA-related diseases with the down-regulated RAGs.
(TIF)

Figure S3 **Gene expression signatures and their enriched cellular processes in PBMCs.** A) A Venn diagram of DEGs depicting the overlap among the DEGs identified from T-cell, B-cell, and PBMCs microarray data. B) GO Biological Processes (GOBPs) enriched by the union of PBMCs signatures (*P*<0.05).
(TIF)

Table S1 **108 molecular target candidates categorized by their associated modules in RA-perturbed network.**
(DOC)

Table S2 **19 key transcription factors categorized by their associated modules in RA-perturbed network.**
(DOC)
