# Supplementary figures and images for: A Systems Approach to Rheumatoid Arthritis
Source: PLoS One. 2012 Dec 11;7(12):e51508. doi: 10.1371/journal.pone.0051508 (PMC3519858; doi:10.1371/journal.pone.0051508)

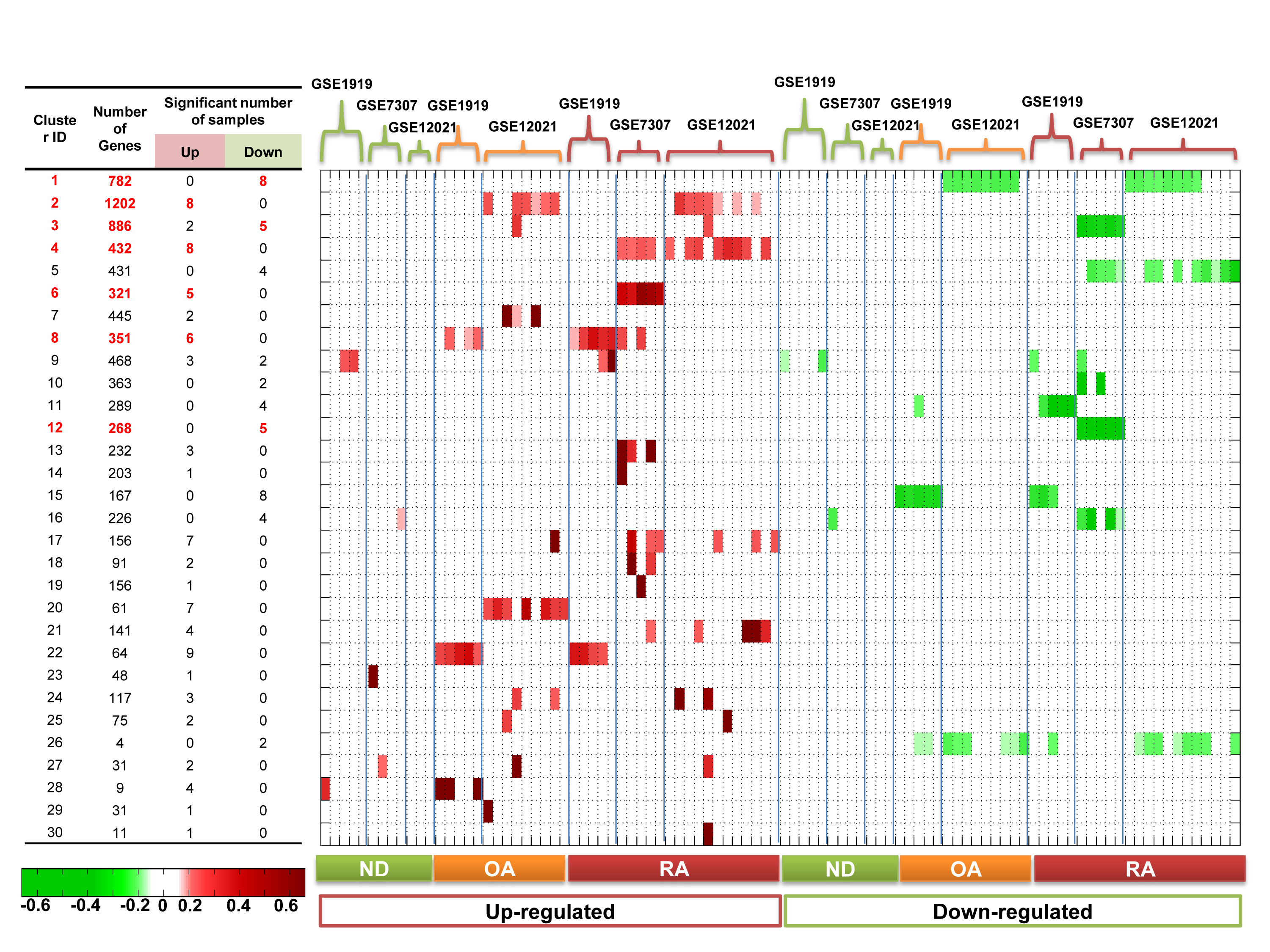

Supplement: Figure S1 — NMF clustering results. (TIF) [file pone.0051508.s001.tif]

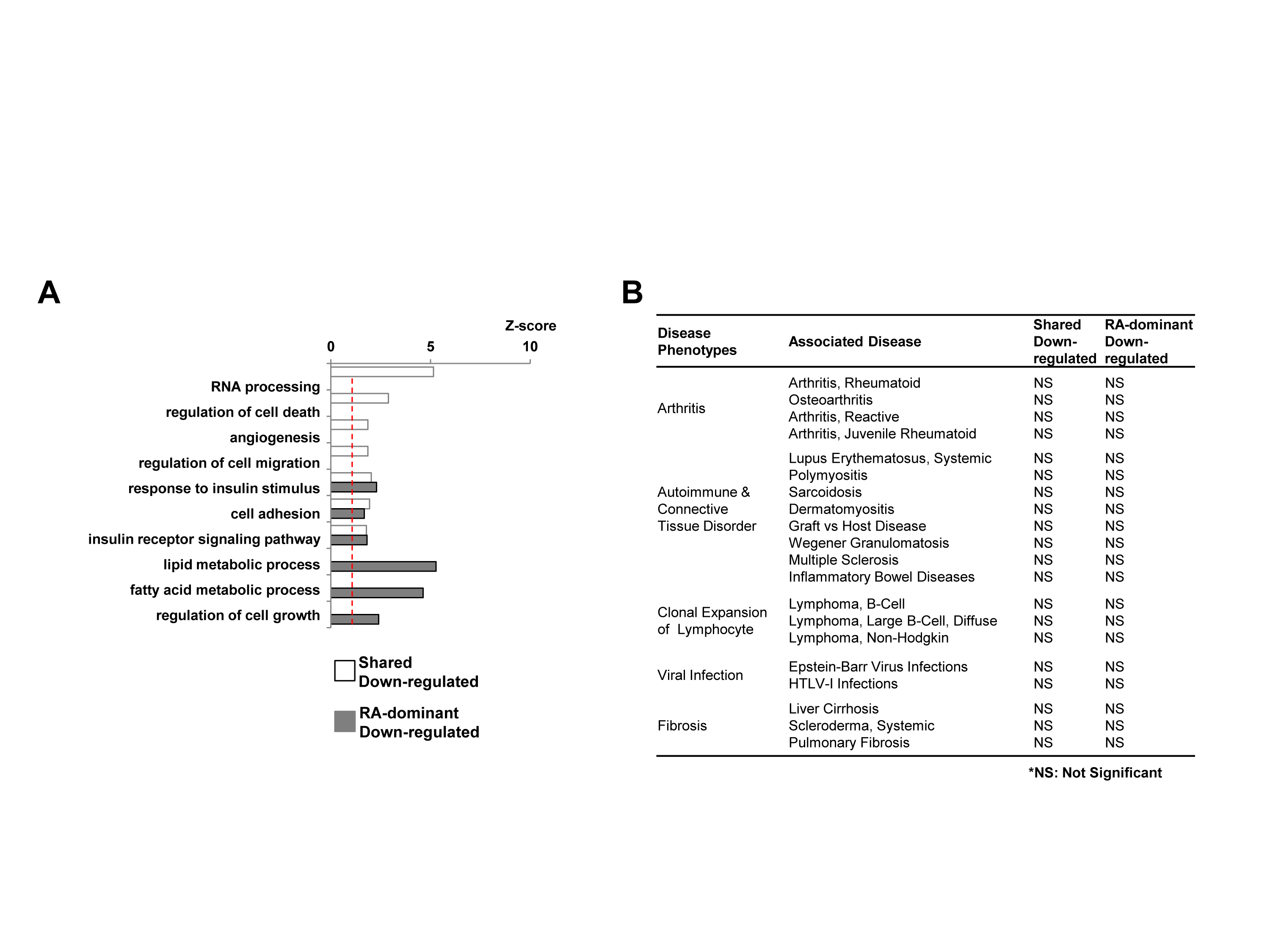

Supplement: Figure S2 — Functional enrichment analysis and disease association analysis for the down-regulated RAGs. A) GOBPs enriched by the shared and RA-dominant down-regulated RAGs (P<0.05). B) Association of five classes of RA-related diseases with the down-regulated RAGs. (TIF) [file pone.0051508.s002.tif]

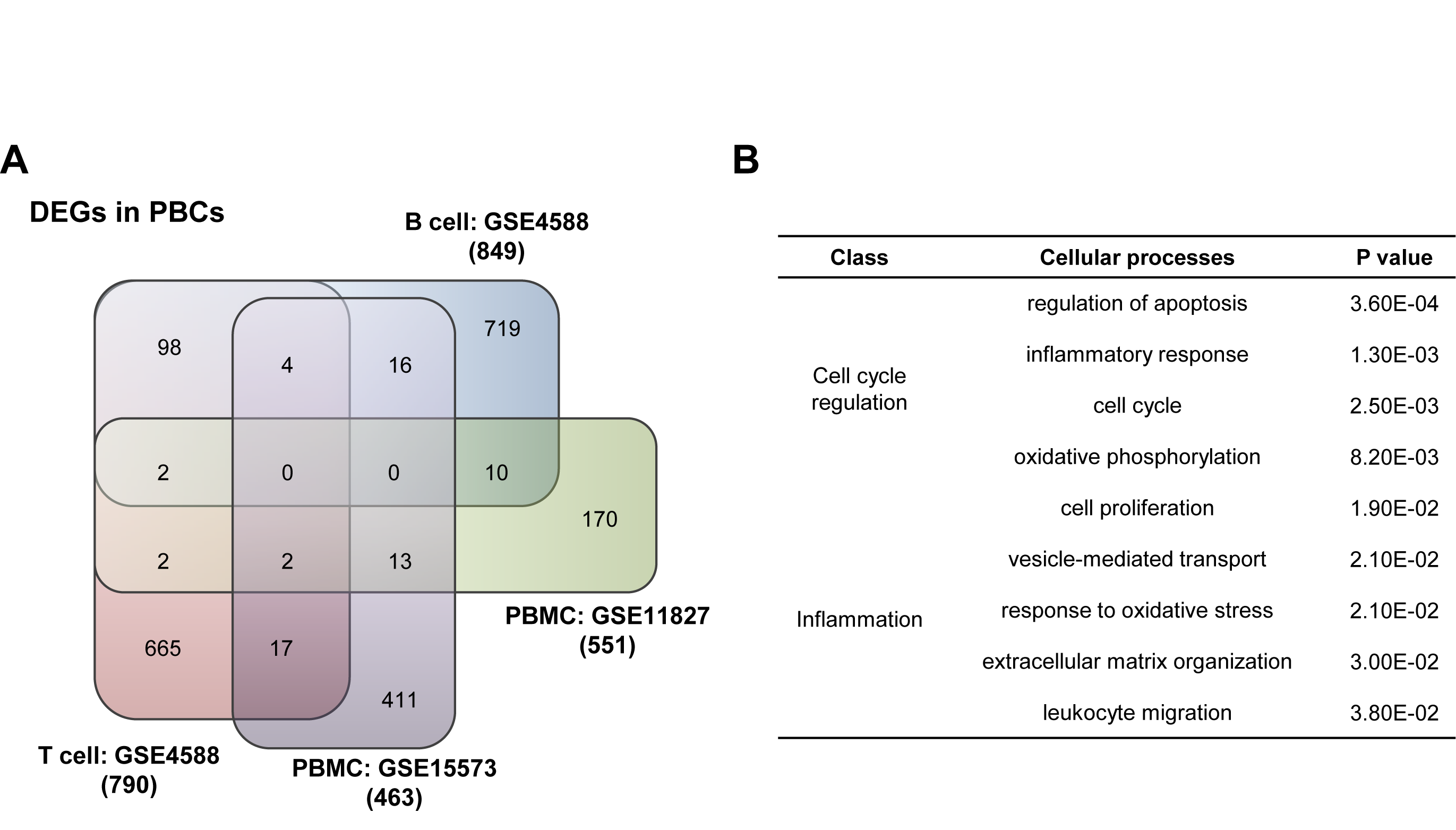

Supplement: Figure S3 — Gene expression signatures and their enriched cellular processes in PBMCs. A) A Venn diagram of DEGs depicting the overlap among the DEGs identified from T-cell, B-cell, and PBMCs microarray data. B) GO Biological Processes (GOBPs) enriched by the union of PBMCs signatures (P<0.05). (TIF) [file pone.0051508.s003.tif]
