# Supplementary material for: A Systems Approach to Rheumatoid Arthritis
Source: PLoS One. 2012 Dec 11;7(12):e51508. doi: 10.1371/journal.pone.0051508 (PMC3519858; doi:10.1371/journal.pone.0051508)
Supplement: Table S1 — 108 molecular target candidates categorized by their associated modules in RA-perturbed network. (DOC) [file pone.0051508.s004.doc]

**Table S1**. 108 molecular target candidates categorized by their associated modules in RA-perturbed network

| **Associated Modules** | **Symbol** | **P Value** | **Chemical** | **Associated Diseases** |
| --- | --- | --- | --- | --- |
| Angiogenic factors | AXL | < 0.00001 | tnf-alpha,tumor necrosis factor,vascular endothelial growth factor,vegf | - |
| Antigen processing and presentation | CIITA | 0.00628 | cortisol | Arthritis, Juvenile Rheumatoid; Arthritis, Rheumatoid; Epstein-Barr Virus Infections; Inflammatory Bowel Diseases; Lupus Erythematosus, Systemic; Lymphoma, B-Cell; Lymphoma, Large B-Cell, Diffuse; Multiple Sclerosis; Wegener Granulomatosis |
| B cell activation | CBL | < 0.00001 | ag 1478,bsab,ci-1033,c-mpl ligand,erlotinib,flt3-ligand,geldanamycin,il-2,imatinib,kinase inhibitor,piceatannol,pma,proteasome inhibitor,ro-31-8220,tgf,thrombin,tyrosine kinase inhibitors,vegf,wortmannin | Lupus Erythematosus, Systemic; Lymphoma, B-Cell; Lymphoma, Non-Hodgkin |
| B cell activation | LYN | < 0.00001 | antioxidant,antiserum,ara-c,bortezomib,bpde,calcitriol,cisplatin,daunorubicin,dnr,egcg,flavopiridol,flt3 ligand,herbimycin,herbimycin a,imatinib,mpa,nystatin,piceatannol,pma,proteasome inhibitor,quercetin,resveratrol,retinoic acid,staurosporine,sti571,taxol,thymidine,tyrosine kinase inhibitor | HTLV-I Infections; Lupus Erythematosus, Systemic; Lymphoma, B-Cell; Lymphoma, Large B-Cell, Diffuse; Lymphoma, Non-Hodgkin |
| B cell activation | PTPN6 | < 0.00001 | antisense oligonucleotides,epinephrine,epo,ida,il-10,il-13,il-2,il-8,interleukin-3,lif,n-acetylcysteine,sms 201-995,tgf | Arthritis, Rheumatoid; Epstein-Barr Virus Infections; HTLV-I Infections; Lupus Erythematosus, Systemic; Lymphoma, B-Cell; Lymphoma, Large B-Cell, Diffuse; Multiple Sclerosis |
| B cell activation | CD79B | < 0.00001 | - | Arthritis, Rheumatoid; Epstein-Barr Virus Infections; Lymphoma, B-Cell; Lymphoma, Large B-Cell, Diffuse; Lymphoma, Non-Hodgkin |
| B cell activation | BLK | < 0.00001 | - | Lupus Erythematosus, Systemic |
| B cell activation | CD19 | < 0.00001 | 5-aza-2'-deoxycytidine,amifostine,cyclosporin a,doxorubicin,hmw-bcgf,il-12,il-6,immunosuppressive,inositol,isoflavone,ppd,retinoid,tcdd,testosterone,thymidine | Arthritis, Rheumatoid; Lupus Erythematosus, Systemic; Lymphoma, B-Cell; Lymphoma, Large B-Cell, Diffuse; Scleroderma, Systemic |
| B cell activation | BTK | < 0.00001 | - | Arthritis, Rheumatoid; Graft vs Host Disease; Lupus Erythematosus, Systemic; Lymphoma, B-Cell; Lymphoma, Non-Hodgkin |
| B cell activation | VAV2 | < 0.00001 | interleukin-2 gene | - |
| B cell activation | RAC1 | < 0.00001 | - | Arthritis, Rheumatoid; HTLV-I Infections; Inflammatory Bowel Diseases; Lupus Erythematosus, Systemic; Multiple Sclerosis |
| B cell activation | CD79A | < 0.00001 | 5-aza-2'-deoxycytidine,5-aza-2-deoxycytidine | Epstein-Barr Virus Infections; Lymphoma, B-Cell; Lymphoma, Large B-Cell, Diffuse; Lymphoma, Non-Hodgkin |
| B cell activation | INPP5D | < 0.00001 | - | Graft vs Host Disease; Lymphoma, B-Cell |
| B cell activation | SLA2 | < 0.00001 | - | - |
| B cell activation | SH2B2 | < 0.00001 | - | - |
| B cell activation | SHB | < 0.00001 | - | - |
| B cell activation | RASA1 | < 0.00001 | apo2l,b3,clonidine,etoposide,thrombin,trail | Lymphoma, Non-Hodgkin |
| B cell activation | ARHGAP17 | < 0.00001 | - | - |
| B cell activation | MS4A2 | < 0.00001 | - | - |
| B cell activation | ARHGDIA | < 0.00001 | - | Inflammatory Bowel Diseases |
| B cell activation | VAV3 | < 0.00001 | - | - |
| B cell activation | PIK3R1 | 0.00001 | - | - |
| B cell activation | SHC1 | 0.00001 | - | Lymphoma, B-Cell; Lymphoma, Large B-Cell, Diffuse; Lymphoma, Non-Hodgkin; Pulmonary Fibrosis |
| B cell activation | PIK3CB | 0.00005 | 2c4,4-hydroxy-tamoxifen,5-fluorouracil,6-hydroxydopamine,6-ohda,activin,albumin,alendronate,androgens,antiangiogenic,anti-cd3 antibody,antiestrogen,anti-inflammatory drugs,antioxidant,atorvastatin,bacillus calmette-guerin,baclofen,basic fibroblast growth factor,bcg,bdnf,bioflavonoid,bpde,c5a,caffeine,camptothecin,carbamazepine,caspase inhibitor,cci-779,cd40l,celecoxib,chemokines,chemopreventive,chemopreventive agent,chondroitin,cilostazol,c-kit ligand,cpt-11,curcumin,cytokines,daidzein,dexamethasone,dhea,dhpg,dmso,dna crosslinking agent,dnr,docetaxel,donepezil,dox,doxorubicin,emodin,etoposide,fas ligand,fdg,flavopiridol,fludarabine,fractalkine,gefitinib,gemcitabine,ginkgo biloba,glucagon,gonadotropins,gp-120,heparin,herceptin,heregulin,hgf,hsp70,hydrogen peroxide,i3c,ici 182,780,ifn,il-13,il-17,il-2,il-5,il-7,il-9,imipramine,indole-3-carbinol,indomethacin,insulin,interferon gamma,intralipid,ionomycin,ip-10,leflunomide,lidocaine,lithium,lovastatin,mannitol,mpa,mtor inhibitor,nelfinavir,neomycin,neurotrophic factor,nordihydroguaiaretic acid,nsaid,nsaids,octreotide,opioid,ox40l,paclitaxel,piceatannol,pkc412,pma,polyphenol,progesterone,progestin,proteasome inhibitor,ps341,quercetin,raloxifene,rantes,ras inhibitor,resveratrol,retinoid,rhil-11,selective estrogen receptor modulator,selenium,serm,simvastatin,sodium butyrate,su5416,sulforaphane,sulindac,suramin,tcdd,testosterone,tgfbeta1,thc,theophylline,thrombin,topoisomerase inhibitor,topoisomerase-ii inhibitor,topotecan,trail,troglitazone,tumor necrosis factor,tyrosine kinase inhibitors,urokinase plasminogen activator,vasopressin,vincristine,wortmannin,zinc sulfate | - |
| B cell activation | PIK3R3 | 0.00007 | - | - |
| B cell activation | PIK3R2 | 0.00008 | - | - |
| B cell activation | HRAS | 0.00009 | aclarubicin,adenoviral vector,adriamycin,anthracyclines,antibiotic,antibiotics,antisense oligonucleotides,antiserum,bb-2516,bortezomib,bpde,bryostatin-1,butyrate,cpt-11,dfmo,doxorubicin,erythropoietin,etoposide,gdnf,gm-csf,hexamethylene bisacetamide,il-3,il-6,inositol,insulin,interleukin-2,interleukin-3,jm-216,m-csf,melphalan,oxaliplatin,oxamflatin,paclitaxel,pd-98059,pirarubicin,protease inhibitor,proteasome inhibitor,protein kinase inhibitor,protein phosphatase inhibitor,r115777,radicicol,rapamycin,ras inhibitor,rbc,sodium butyrate,sulfone,sulindac sulfide,tamoxifen,tcdd,tgfbeta1,thrombin,thymidine,toremifene,ucn-01,viral vector,wortmannin | Arthritis, Rheumatoid; Liver Cirrhosis; Lupus Erythematosus, Systemic; Lymphoma, B-Cell; Lymphoma, Non-Hodgkin; Osteoarthritis; Pulmonary Fibrosis; Scleroderma, Systemic |
| B cell activation | VAV1 | 0.00014 | cytokines,epo,erythropoietin,g-csf,gm-csf,granulocyte macrophage colony-stimulating factor,hmba,ifn,il-2,il-4,il-5,il-6,interleukin-2,interleukin-3,m-csf,methylcellulose,pioglitazone,pma,troglitazone,tumor necrosis factor | HTLV-I Infections; Lupus Erythematosus, Systemic; Lymphoma, B-Cell; Lymphoma, Large B-Cell, Diffuse |
| B cell activation | PRKCD | 0.00044 | kinase inhibitor,uvb | Lupus Erythematosus, Systemic; Lymphoma, Large B-Cell, Diffuse; Pulmonary Fibrosis; Scleroderma, Systemic |
| B cell activation | PRKCB | 0.00208 | 12-o-tetradecanoylphorbol-13-acetate,antisense oligonucleotides,celecoxib,dag,d-alpha-tocopherol,desferrioxamine,dexamethasone,hmba,ionomycin,lisinopril,phorbol myristate acetate,pma,protein phosphatase inhibitor,retinoic acid,thrombin,tpa | Arthritis, Rheumatoid; Inflammatory Bowel Diseases; Lymphoma, B-Cell; Lymphoma, Large B-Cell, Diffuse |
| Cell cycle | KHDRBS1 | < 0.00001 | cobalt,kinase inhibitor,radicicol | HTLV-I Infections |
| Cell cycle | MAPK3 | 0.00026 | 13-cis-retinoic acid,2'-amino-3'-methoxyflavone,5'-deoxy-5-fluorouridine,5-fu,6-ohda,8-cl-camp,9-cis-retinoic acid,actinomycin d,adriamycin,ag 1478,alpha-difluoromethylornithine,alpha-tocopherol,amd3100,amphetamine,antibiotics,antiestrogen,antiestrogens,anti-inflammatory drugs,antineoplastic,antiserum,anti-vegf,arsenic trioxide,ascorbic acid,aspirin,atenolol,atorvastatin,azaserine,batimastat,bcg,bortezomib,bpde,bryostatin-1,bso,bumetanide,c225,c5a,caffeine,calcitonin,calcitriol,camptothecin,carbamazepine,carvedilol,cd40l,cda,cetuximab,chelators,chemopreventive,chemoprotective,chemotherapeutic agent,chondroitin,cisplatin,clofibrate,cobalt,colchicine,cyclophosphamide,cytokines,dfmo,dht,digitoxin,digoxin,dipyridamole,dnr,docetaxel,dox,doxycycline,echinomycin,egfr tyrosine kinase inhibitors,ekb-569,emodin,eotaxin,epinephrine,epirubicin,epo,erbitux,erlotinib,erythropoietin,etidronate,etoposide,farnesyl transferase inhibitor,fas ligand,fas-ligand,folic acid,fractalkine,fulvestrant,g-csf,gefitinib,geldanamycin,gemcitabine,genistein,glucagon,glucocorticoid,glyburide,gm-csf,gonadotropins,gp-120,green tea extract,gw572016,harp,heparan sulfate,heregulin,hgf,human chorionic gonadotropin,hypericin,ifn,il-11,il-17,il-18,il-1ra,il-3,il-4,il-5,il-6,il-7,il-8,immunosuppressive,indomethacin,insulin,ip-10,irbesartan,irofulven,isoflavone,kinase inhibitor,l-744,832,latanoprost,lovastatin,l-thyroxine,ly293111,mannitol,mapk inhibitors,marimastat,matrix metalloproteinase inhibitor,melphalan,mip-1beta,mistletoe lectin,mmp inhibitor,moxifloxacin,mpa,naloxone,nandrolone,ndga,nicotine,nifedipine,nitroglycerin,nonsteroidal anti-inflammatory drugs,opiate,paclitaxel,pamidronate,pd-168393,pd-98059,peitc,phenylephrine,photofrin,phytochemical,piceatannol,pki166,pma,pravastatin,prazosin,progestin,propranolol,protease inhibitor,proteasome inhibitor,protein kinase a inhibitor,protein kinase c inhibitor,protein kinase inhibitor,protein phosphatase inhibitor,protoporphyrin,puromycin,quercetin,r115777,radicicol,rantes,ras inhibitor,retinoic acid,retinoid,retinol,risedronate,risperidone,ritonavir,rituximab,roxithromycin,rtx,saha,salmeterol,selenium,silymarin,sodium salicylate,sorbitol,soy isoflavone,spironolactone,sr49059,steel factor,su5402,su5416,su6668,sulforaphane,sulindac,sulindac sulfide,suramin,tam,tamoxifen,tcdd,tetrahydrocannabinol,tgf-beta 2,tgfbeta1,thc,tnf-alpha,trail,trapoxin,triptorelin,troglitazone,tumor antigen,tyrosine kinase inhibitors,tyrphostin a9,ucn-01,urea,urokinase,vcr,verapamil,vincristine,vitamin d,wortmannin,zd6474,zncl2,zoledronate | Scleroderma, Systemic |
| Cell cycle | FZR1 | 0.00546 | - | - |
| Cell daath and survival | CFLAR | 0.00139 | - | Arthritis, Rheumatoid; Lupus Erythematosus, Systemic; Lymphoma, B-Cell; Lymphoma, Large B-Cell, Diffuse; Lymphoma, Non-Hodgkin; Multiple Sclerosis; Osteoarthritis; Polymyositis; Pulmonary Fibrosis |
| Cell daath and survival | CASP10 | 0.00401 | - | Arthritis, Rheumatoid; Graft vs Host Disease; Lymphoma, Non-Hodgkin |
| Cell death and survival | FASLG | < 0.00001 | 4-hc,4-hydroperoxycyclophosphamide,5-aza-2'-deoxycytidine,5-fluorouracil,5-fu,abt-510,actinomycin d,alpha-difluoromethylornithine,androgens,antibiotic,anti-cd3 antibody,antiestrogens,aplidin,arsenic trioxide,beta-lapachone,bortezomib,bso,capecitabine,carmustine,ccnu,cell surface antigen,cisplatin,cortisol,ctx,curcumin,cyclophosphamide,cytokines,deoxycytidine,dfmo,doxorubicin,doxycycline,dtic,egcg,epo,erythropoietin,ether,fas-ligand,fr901228,ganciclovir,glucocorticoid,gossypol,gp-120,hel,hen egg lysozyme,hoechst 33258,ifn,il-17,il-3,imexon,lymphokine-activated killer cells,lymphokines,mart-1,m-csf,mistletoe lectin,ok-432,opioid,poly i:c,polyphenol,protease inhibitors,protein phosphatase inhibitor,protein synthesis inhibitor,psoralen,resveratrol,saponin,sodium butyrate,testosterone undecanoate,tetracycline,tetrandrine,thymosin,tnf-alpha,topotecan,trail,treosulfan,tumor antigen,tumor necrosis factor,tumor necrosis factor-beta,tyrosine kinase inhibitor,vincristine | Arthritis, Rheumatoid; Dermatomyositis; Epstein-Barr Virus Infections; Graft vs Host Disease; HTLV-I Infections; Inflammatory Bowel Diseases; Liver Cirrhosis; Lupus Erythematosus, Systemic; Lymphoma, B-Cell; Lymphoma, Large B-Cell, Diffuse; Lymphoma, Non-Hodgkin; Multiple Sclerosis; Osteoarthritis; Polymyositis; Pulmonary Fibrosis; Scleroderma, Systemic; Wegener Granulomatosis |
| Cell death and survival | DOK1 | < 0.00001 | - | Epstein-Barr Virus Infections |
| Cell death and survival | TNFRSF1A | < 0.00001 | actinomycin d,antibiotic,antioxidant,autoantigen,bb-2516,bryostatin-1,c5a,ceftriaxone,chloramphenicol,corticotropin,dmso,ethanol,fludarabine,geldanamycin,h2o2,hydrogen peroxide,indomethacin,interferon gamma,ionomycin,leflunomide,mmf,mtx,ndga,nicotine,nsaids,pentoxifylline,ppd,protease inhibitor,proteasome inhibitor,protein kinase c inhibitor,saha,sch 66336,sch-66336,sodium salicylate,staurosporine,tetrahydrocannabinol,thalidomide,uvb,zdv,zidovudine | Arthritis, Juvenile Rheumatoid; Arthritis, Rheumatoid; Graft vs Host Disease; HTLV-I Infections; Inflammatory Bowel Diseases; Liver Cirrhosis; Lupus Erythematosus, Systemic; Lymphoma, B-Cell; Lymphoma, Non-Hodgkin; Multiple Sclerosis; Osteoarthritis; Wegener Granulomatosis |
| Cell death and survival | DOK2 | < 0.00001 | - | Lymphoma, B-Cell |
| Cell death and survival | PRKCD | 0.00044 | kinase inhibitor,uvb | Lupus Erythematosus, Systemic; Lymphoma, Large B-Cell, Diffuse; Pulmonary Fibrosis; Scleroderma, Systemic |
| Cell death and survival | FADD | 0.00094 | trail | Arthritis, Rheumatoid; Lupus Erythematosus, Systemic; Lymphoma, B-Cell; Pulmonary Fibrosis |
| Cell death and survival | TRADD | 0.00273 | sch-66336 | Liver Cirrhosis; Lupus Erythematosus, Systemic; Lymphoma, Non-Hodgkin |
| Cell death and survival | HMGB1 | 0.00407 | adm,antisense oligonucleotides,antiviral,carboplatin,fludarabine,glycyrrhizin,hormones,jm-216,nicotine,oxaliplatin,pd-98059,progesterone,retinoic acid,steroid hormone,tgfbeta1,t-pa | Arthritis, Rheumatoid; Dermatomyositis; Lymphoma, Non-Hodgkin; Multiple Sclerosis; Osteoarthritis; Pulmonary Fibrosis |
| Cell migration and adhesion | FYN | < 0.00001 | 12-o-tetradecanoylphorbol-13-acetate,anti-cd3 antibody,antisense oligonucleotides,antiserum,autologous tumor cells,bdnf,colchicine,cyclosporin a,dexamethasone,donepezil,ethanol,fas ligand,gdnf,geldanamycin,genistein,gp-120,herbimycin a,hgf,ifn,il-11,il-2,il-2 gene,il-3,il-4,il-6,inositol,insulin,interleukin-11,ionomycin,kinase inhibitor,leflunomide,m-csf,nsaid,nsaids,pma,quercetin,staurosporine,thrombin,tnf-alpha,tpa,transforming growth factor,tyrosine kinase inhibitors,uvb,vegf | Arthritis, Rheumatoid; Epstein-Barr Virus Infections; HTLV-I Infections; Liver Cirrhosis; Lupus Erythematosus, Systemic; Lymphoma, B-Cell; Pulmonary Fibrosis |
| Cell migration and adhesion | CXCR4 | < 0.00001 | amd3100,chemokines | Arthritis, Rheumatoid; Dermatomyositis; Epstein-Barr Virus Infections; Graft vs Host Disease; Lupus Erythematosus, Systemic; Lymphoma, B-Cell; Lymphoma, Non-Hodgkin; Osteoarthritis; Scleroderma, Systemic |
| Cell migration and adhesion | CXCR2 | < 0.00001 | mgsa,proteasome inhibitor | Arthritis, Rheumatoid; Lupus Erythematosus, Systemic; Lymphoma, Large B-Cell, Diffuse; Multiple Sclerosis; Osteoarthritis |
| Cell migration and adhesion | CD44 | < 0.00001 | adjuvant,antiangiogenic,atg,atgam,blm,bnct agent,bovine cartilage,butyrate,c5a,cisplatin,daunorubicin,dexamethasone,dnr,dx-52-1,etoposide,g-csf,genistein,gp-120,herbimycin a,hgf,il-18,il-2,il-6,il-8,interferon gamma,ionomycin,isoproterenol,kinase inhibitor,mip-1 beta,mip-1beta,mitoxantrone,pd-98059,pentoxifylline,phosphorothioate oligonucleotide,polyinosinic acid:polycytidylic acid,rantes,rna polymerase inhibitor,tgfbeta1,thymoglobulin,tnf-alpha,tumor necrosis factor,tyrosine kinase inhibitor | Arthritis, Rheumatoid; Graft vs Host Disease; Lupus Erythematosus, Systemic; Lymphoma, B-Cell; Lymphoma, Large B-Cell, Diffuse; Lymphoma, Non-Hodgkin; Multiple Sclerosis; Osteoarthritis |
| Cell migration and adhesion | RAC1 | < 0.00001 | - | Arthritis, Rheumatoid; HTLV-I Infections; Inflammatory Bowel Diseases; Lupus Erythematosus, Systemic; Multiple Sclerosis |
| Cell migration and adhesion | WASF2 | < 0.00001 | - | - |
| Cell migration and adhesion | WAS | < 0.00001 | - | Epstein-Barr Virus Infections; Graft vs Host Disease; Inflammatory Bowel Diseases; Lymphoma, B-Cell; Lymphoma, Large B-Cell, Diffuse; Lymphoma, Non-Hodgkin; Pulmonary Fibrosis |
| Cell migration and adhesion | ARHGAP17 | < 0.00001 | - | - |
| Cell migration and adhesion | ARHGDIA | < 0.00001 | - | Inflammatory Bowel Diseases |
| Cell migration and adhesion | EPHB1 | < 0.00001 | - | Arthritis, Rheumatoid; Multiple Sclerosis |
| Cell migration and adhesion | INSR | < 0.00001 | ethanol,glipizide | - |
| Cell migration and adhesion | VAV3 | < 0.00001 | - | - |
| Cell migration and adhesion | PLAUR | < 0.00001 | adenoviral vector,adenovirus vector,antisense oligonucleotides,c5a,chelators,colchicine,cytokines,herbimycin,il-1,il-4,iressa,nystatin,pd-98059,rapamycin,somatomedin,tamoxifen,thalidomide,tnf-alpha,tpa,tyrosine kinase inhibitors,urokinase-type plasminogen activator | Arthritis, Reactive; Arthritis, Rheumatoid; Liver Cirrhosis; Lymphoma, Large B-Cell, Diffuse; Multiple Sclerosis; Osteoarthritis; Pulmonary Fibrosis; Scleroderma, Systemic |
| Cell migration and adhesion | PIK3R1 | 0.00001 | - | - |
| Cell migration and adhesion | SHC1 | 0.00001 | - | Lymphoma, B-Cell; Lymphoma, Large B-Cell, Diffuse; Lymphoma, Non-Hodgkin; Pulmonary Fibrosis |
| Cell migration and adhesion | IRS2 | 0.00004 | aspirin,epo,erythropoietin,ifn,il-13,il-2,il-7,insulin,nsaids,progesterone,rosiglitazone,tgfbeta1,tnf-alpha | Lymphoma, B-Cell |
| Cell migration and adhesion | PIK3CB | 0.00005 | 2c4,4-hydroxy-tamoxifen,5-fluorouracil,6-hydroxydopamine,6-ohda,activin,albumin,alendronate,androgens,antiangiogenic,anti-cd3 antibody,antiestrogen,anti-inflammatory drugs,antioxidant,atorvastatin,bacillus calmette-guerin,baclofen,basic fibroblast growth factor,bcg,bdnf,bioflavonoid,bpde,c5a,caffeine,camptothecin,carbamazepine,caspase inhibitor,cci-779,cd40l,celecoxib,chemokines,chemopreventive,chemopreventive agent,chondroitin,cilostazol,c-kit ligand,cpt-11,curcumin,cytokines,daidzein,dexamethasone,dhea,dhpg,dmso,dna crosslinking agent,dnr,docetaxel,donepezil,dox,doxorubicin,emodin,etoposide,fas ligand,fdg,flavopiridol,fludarabine,fractalkine,gefitinib,gemcitabine,ginkgo biloba,glucagon,gonadotropins,gp-120,heparin,herceptin,heregulin,hgf,hsp70,hydrogen peroxide,i3c,ici 182,780,ifn,il-13,il-17,il-2,il-5,il-7,il-9,imipramine,indole-3-carbinol,indomethacin,insulin,interferon gamma,intralipid,ionomycin,ip-10,leflunomide,lidocaine,lithium,lovastatin,mannitol,mpa,mtor inhibitor,nelfinavir,neomycin,neurotrophic factor,nordihydroguaiaretic acid,nsaid,nsaids,octreotide,opioid,ox40l,paclitaxel,piceatannol,pkc412,pma,polyphenol,progesterone,progestin,proteasome inhibitor,ps341,quercetin,raloxifene,rantes,ras inhibitor,resveratrol,retinoid,rhil-11,selective estrogen receptor modulator,selenium,serm,simvastatin,sodium butyrate,su5416,sulforaphane,sulindac,suramin,tcdd,testosterone,tgfbeta1,thc,theophylline,thrombin,topoisomerase inhibitor,topoisomerase-ii inhibitor,topotecan,trail,troglitazone,tumor necrosis factor,tyrosine kinase inhibitors,urokinase plasminogen activator,vasopressin,vincristine,wortmannin,zinc sulfate | - |
| Cell migration and adhesion | PIK3R3 | 0.00007 | - | - |
| Cell migration and adhesion | PIK3R2 | 0.00008 | - | - |
| Cell migration and adhesion | VAV1 | 0.00014 | cytokines,epo,erythropoietin,g-csf,gm-csf,granulocyte macrophage colony-stimulating factor,hmba,ifn,il-2,il-4,il-5,il-6,interleukin-2,interleukin-3,m-csf,methylcellulose,pioglitazone,pma,troglitazone,tumor necrosis factor | HTLV-I Infections; Lupus Erythematosus, Systemic; Lymphoma, B-Cell; Lymphoma, Large B-Cell, Diffuse |
| Chemokines | CXCR4 | < 0.00001 | amd3100,chemokines | Arthritis, Rheumatoid; Dermatomyositis; Epstein-Barr Virus Infections; Graft vs Host Disease; Lupus Erythematosus, Systemic; Lymphoma, B-Cell; Lymphoma, Non-Hodgkin; Osteoarthritis; Scleroderma, Systemic |
| Chemokines | CSF2RB | < 0.00001 | - | - |
| Chemokines | CXCR2 | < 0.00001 | mgsa,proteasome inhibitor | Arthritis, Rheumatoid; Lupus Erythematosus, Systemic; Lymphoma, Large B-Cell, Diffuse; Multiple Sclerosis; Osteoarthritis |
| Chemokines | PDGFB | < 0.00001 | 12-o-tetradecanoylphorbol-13-acetate,isoproterenol,pma,prostaglandin e1,protein kinase inhibitor,thrombin,tpa | Liver Cirrhosis; Lymphoma, Non-Hodgkin; Multiple Sclerosis; Scleroderma, Systemic |
| Chemokines | CCR3 | 0.00001 | cc chemokines,chemokines,distamycin,eotaxin,heparin,il-16,il-4,il-5,rantes,staurosporine,thymidine,transforming growth factor | Arthritis, Juvenile Rheumatoid; Arthritis, Rheumatoid; Liver Cirrhosis; Lymphoma, B-Cell; Lymphoma, Large B-Cell, Diffuse; Lymphoma, Non-Hodgkin; Multiple Sclerosis; Osteoarthritis; Pulmonary Fibrosis; Wegener Granulomatosis |
| ECM organization | COL1A2 | < 0.00001 | genistein,ifn,il-4,imatinib mesylate,interferon gamma,simvastatin,tgf,tgf-beta 2,tgfbeta1,tyrosine kinase inhibitor | Multiple Sclerosis; Scleroderma, Systemic |
| ECM organization | RELN | < 0.00001 | deoxycytidine,suberoylanilide hydroxamic acid,valproic acid | Liver Cirrhosis |
| Fc epsilon RI signaling | INPP5D | < 0.00001 | - | Graft vs Host Disease; Lymphoma, B-Cell |
| Fc gamma receptor mediated phagocytosis | INPP5D | < 0.00001 | - | Graft vs Host Disease; Lymphoma, B-Cell |
| Inflammatory cytokines | KIT | < 0.00001 | all-trans retinoic acid,cyclosporin a,dmso,gleevec,imatinib,imatinib mesylate,mast cell growth factor,steel factor,tpa,tyrosine kinase inhibitor | Arthritis, Rheumatoid; Inflammatory Bowel Diseases; Lymphoma, B-Cell; Lymphoma, Large B-Cell, Diffuse |
| Inflammatory cytokines | IL2RB | < 0.00001 | - | Arthritis, Rheumatoid; HTLV-I Infections |
| Inflammatory cytokines | CSF3R | < 0.00001 | - | Multiple Sclerosis |
| Inflammatory cytokines | IL2 | < 0.00001 | 5-fluoro-2'-deoxyuridine,8-methoxypsoralen,actinomycin d,alendronate,alpha-methyl-p-tyrosine,alvac,anesthetics,anthracycline,anti-cd23,antihistamine,antimalarial drugs,antioxidant,antiserum,antitumor antibiotic,apo2l,atg,atropine,bacillus calmette-guerin,bcg vaccine,bcx-1777,bcx-34,budesonide,buspirone,capsaicin,caspase inhibitor,c-c chemokines,celecoxib,chinese herbs,clozapine,c-myb antisense,cobalt,cqs,c-raf antisense,csa,curcumin,d4t,ddd,differentiating agents,dihydrosphingosine,dipyridamole,dna polymerase inhibitor,dnr,egcg,emodin,epigallocatechin gallate,et-18-och3,filgrastim,fractalkine,garlic,garlic extract,geldanamycin,genistein,ginger,glucagon,herbimycin a,histamine dihydrochloride,hpv-16 e6,human chorionic gonadotropin,ifosfamide,il-4,imipenem,imipramine,indomethacin,influenza vaccine,interferon beta-1b,intralipid,ionomycin,isoniazid,itraconazole,ketoconazole,l-778,123,lak,lak cells,low molecular weight heparin,mafosfamide,marijuana,mcp-2,methylprednisolone,mgn3,mip-1 alpha,monoclonal antibody 17-1a,mtx,muc-1,muc1 mucin,multivitamin,naloxone,ofloxacin,ondansetron,opioid antagonist,oral contraceptive,ox40l,oxaliplatin,paracetamol,penicillamine,phenothiazine,phenylacetate,phosphodiesterase inhibitor,photosensitizer,phytochemicals,piceatannol,prednisone,propionibacterium acnes,propofol,proteasome inhibitor,protein synthesis inhibitor,protoporphyrin,quinine,r24,radicicol,rcd4,recombinant transforming growth factor-beta,reserpine,rhgh,risperidone,ritonavir,rituximab,ro 31-8220,salmeterol,simvastatin,sodium salicylate,spironolactone,steroid hormone,suberoylanilide hydroxamic acid,sulfasalazine,suramin,taurolidine,terfenadine,tetrandrine,thalidomide,tnf-related apoptosis-inducing ligand,trail,tramadol,tributyrin,trichostatin a,trifluoperazine,tropisetron,tumor necrosis factor,tyrosinase peptide,ursodeoxycholic acid,valproic acid,vasopressin,verapamil,viscum | Arthritis, Juvenile Rheumatoid; Arthritis, Rheumatoid; Epstein-Barr Virus Infections; Graft vs Host Disease; HTLV-I Infections; Inflammatory Bowel Diseases; Lupus Erythematosus, Systemic; Lymphoma, Large B-Cell, Diffuse; Lymphoma, Non-Hodgkin; Multiple Sclerosis; Osteoarthritis; Pulmonary Fibrosis; Sarcoidosis; Scleroderma, Systemic |
| Inflammatory cytokines | IL4 | < 0.00001 | - | Arthritis, Juvenile Rheumatoid; Arthritis, Rheumatoid; Dermatomyositis; Graft vs Host Disease; Inflammatory Bowel Diseases; Liver Cirrhosis; Lupus Erythematosus, Systemic; Lymphoma, B-Cell; Lymphoma, Large B-Cell, Diffuse; Lymphoma, Non-Hodgkin; Multiple Sclerosis; Osteoarthritis; Polymyositis; Pulmonary Fibrosis; Scleroderma, Systemic |
| Inflammatory cytokines | IL6ST | < 0.00001 | proteasome inhibitor,ps-341 | Arthritis, Juvenile Rheumatoid; Arthritis, Rheumatoid; Inflammatory Bowel Diseases; Liver Cirrhosis; Lymphoma, B-Cell; Multiple Sclerosis; Pulmonary Fibrosis; Scleroderma, Systemic |
| Inflammatory cytokines | IL2RG | < 0.00001 | - | Arthritis, Rheumatoid; Graft vs Host Disease; HTLV-I Infections; Lymphoma, B-Cell |
| Inflammatory cytokines | IL4R | < 0.00001 | csa,il-13,il-4,staurosporine | Arthritis, Juvenile Rheumatoid; Arthritis, Rheumatoid; Graft vs Host Disease; Inflammatory Bowel Diseases; Liver Cirrhosis; Lupus Erythematosus, Systemic; Lymphoma, B-Cell; Lymphoma, Large B-Cell, Diffuse; Lymphoma, Non-Hodgkin; Multiple Sclerosis; Osteoarthritis; Sarcoidosis |
| Inflammatory cytokines | EPOR | < 0.00001 | cytokines | - |
| Inflammatory cytokines | TNF | < 0.00001 | 2-methoxyestradiol,4-hpr,5-aza-2'-deoxycytidine,5'-deoxy-5-fluorouridine,9-nitrocamptothecin,aclacinomycin,adenosine deaminase inhibitor,alum,anti-androgen,anticachexia,antiestrogen,antihypertensive drugs,antiinflammatory drugs,antimalarial drugs,antisense oligonucleotides,anti-tac,anti-vegf,atenolol,atorvastatin,aztreonam,beta-lapachone,bioflavonoid,budesonide,bupivacaine,butein,butylated hydroxytoluene,butyrate,c5a,calcium channel blocker,capsaicin,carbamazepine,carboplatin,cc-5013,cefotaxime,ceftazidime,chromomycin a3,cisplatin,cobalt,cortisone,cyproterone,cytoprotective agent,daidzein,daunomycin,diazoxide,diethyldithiocarbamate,digitoxin,dihydrosphingosine,dimethyl sulfoxide,ell-12,enoxaparin,epothilone b,et-18-och3,etidronate,famotidine,fas ligand,fas-l,fas-ligand,fenretinide,fostriecin,ganciclovir,gatifloxacin,gd-dtpa,gefitinib,gene therapy vectors,glyburide,gm-csf,gonadotropins,hexamethylene bisacetamide,histone deacetylase inhibitor,hpv-16 e6,human chorionic gonadotropin,hydroxamic acids,hydroxyurea,ici 182,780,idoxifene,igiv,il-18,il-2,imipramine,immune adjuvant,indole-3-carbinol,insulin,intercalator,interferon beta-1b,interferons,isoflavone,ketoconazole,labetalol,lactose,lavendustin a,lentinan,leucovorin,lisofylline,lithium,lmwh,lt-beta,lymphotoxin beta,mannose-binding lectin,marijuana,medroxyprogesterone,meg,mesalamine,methimazole,methylene blue,mgsa,mip-1 alpha,mithramycin,mitomycin,mmf,monoclonal antibody okt3,muramyl tripeptide phosphatidylethanolamine,neomycin,new agent,nicardipine,nimodipine,nitrogen mustard,oral contraceptive,oxaliplatin,p30,pala,penicillamine,phenothiazine,phenoxodiol,phosphorothioate oligonucleotide,polyphenol,prazosin,propofol,protein phosphatase inhibitor,protein synthesis inhibitor,psc-833,pyridoxine,reserpine,resiniferatoxin,resveratrol,rifabutin,saha,salt,sandoglobulin,saponin,saquinavir,signal transduction inhibitors,silymarin,simvastatin,sodium salicylate,soy isoflavone,sulindac,sulindac sulfide,suramin,tag-72,tcdd,terbinafine hydrochloride,thalidomide,tnf-related apoptosis-inducing ligand,topotecan,toremifene,tpa,trail,triamcinolone,trifluoperazine,tropisetron,u-0126,urea,uvb,vaccine,vesnarinone,vincristine,vitamin c,vm-26,wortmannin | Arthritis, Juvenile Rheumatoid; Arthritis, Reactive; Arthritis, Rheumatoid; Dermatomyositis; Epstein-Barr Virus Infections; Graft vs Host Disease; HTLV-I Infections; Inflammatory Bowel Diseases; Liver Cirrhosis; Lupus Erythematosus, Systemic; Lymphoma, B-Cell; Lymphoma, Large B-Cell, Diffuse; Lymphoma, Non-Hodgkin; Multiple Sclerosis; Osteoarthritis; Polymyositis; Pulmonary Fibrosis; Sarcoidosis; Scleroderma, Systemic; Wegener Granulomatosis |
| Inflammatory cytokines | IL6 | < 0.00001 | 2me2,4-hc,5-aza-2'-deoxycytidine,6-mercaptopurine,6-mp,activin,allogeneic lymphocytes,amiodarone,antiparasitic,autoantigen,bicalutamide,cancer vaccine,casodex,cefotaxime,cgp41251,chamomile,clomiphene citrate,cortisone,dac,dalteparin,diphtheria toxin,doxorubicin,enalapril,genistein,hmw-bcgf,hydroxyurea,il-17,il-18,inositol,insulin,interferon alpha-2b,ionomycin,iudr,loperamide,macrolides,melanoma vaccine,mito,mmf,oestrogens,oxaliplatin,pd-184352,polyethylene glycol,propofol,ps-341,rhil-4,staurosporine,taxol,teicoplanin,tumor antigen,vincristine,zebularine,zileuton,zinc oxide | Arthritis, Juvenile Rheumatoid; Arthritis, Reactive; Arthritis, Rheumatoid; Epstein-Barr Virus Infections; Graft vs Host Disease; HTLV-I Infections; Inflammatory Bowel Diseases; Liver Cirrhosis; Lupus Erythematosus, Systemic; Lymphoma, B-Cell; Lymphoma, Large B-Cell, Diffuse; Lymphoma, Non-Hodgkin; Multiple Sclerosis; Osteoarthritis; Pulmonary Fibrosis; Sarcoidosis; Scleroderma, Systemic; Wegener Granulomatosis |
| Inflammatory cytokines | IL8 | 0.00001 | 2b1,2-chlorodeoxyadenosine,acetazolamide,alendronate,alpha interferon,aluminum hydroxide,amb,amd3100,amoxicillin-clavulanic acid,amphotericin b,anesthetics,anthralin,antidepressants,antifungal agent,atorvastatin,azathioprine,bcg vaccine,bestatin,biochanin a,bso,ca19-9,calcitriol,capsaicin,carbonic anhydrase inhibitor,cc chemokines,cd40l,cda,cetuximab,chelating agent,chelators,cladribine,cyclooxygenase inhibitor,cyclophosphamide,dapsone,digitoxin,dncb,egfr inhibitor,enzyme inhibitor,etretinate,famotidine,fentanyl,filgrastim,fludarabine,folic acid,fudr,gdnf,glutamine,glycyrrhetinic acid,gp-120,herbimycin a,i3c,ibuprofen,il-13,il-17,indomethacin,isoflavone,ketoconazole,lansoprazole,lavendustin a,leflunomide,lidocaine,lt-beta,lym-1,macrolide antibiotic,mdx-h210,mesalamine,methimazole,midazolam,mifepristone,nac,ndga,nordihydroguaiaretic acid,nystatin,octreotide,ofloxacin,omeprazole,pamidronate,pheniramine maleate,phenoxodiol,phytoestrogen,piceatannol,polyethylene glycol,povidone iodine,povidone-iodine,prednisolone,progesterone,protective agent,protein synthesis inhibitor,rituximab,ro 31-8220,rosiglitazone,roxithromycin,signal transduction inhibitors,sulfasalazine,suramin,talc,tcdd,teicoplanin,tetracycline,thymidine,tnfr:fc,trichostatin a,troglitazone,tyrosine kinase inhibitor,tyrphostin a9,u-0126,uridine,uvb,verapamil,wortmannin | Arthritis, Juvenile Rheumatoid; Arthritis, Rheumatoid; Graft vs Host Disease; Inflammatory Bowel Diseases; Lupus Erythematosus, Systemic; Lymphoma, B-Cell; Lymphoma, Large B-Cell, Diffuse; Lymphoma, Non-Hodgkin; Multiple Sclerosis; Osteoarthritis; Scleroderma, Systemic; Wegener Granulomatosis |
| Inflammatory cytokines | TNFSF10 | 0.00021 | 2me2,5-aza-2'-deoxycytidine,acetylsalicylic acid,actinomycin d,adjuvant,adriamycin,albumin,androgens,anthracycline,antibiotic,antioxidant,antisense oligonucleotides,antiserum,arsenic trioxide,asa,ascorbic acid,azidothymidine,azt,bortezomib,camptosar,caspase inhibitor,ccnu,chemopreventive,cisplatin,cpt-11,curcumin,cyclophosphamide,cyclosporin a,dht,dmso,doxorubicin,egcg,epirubicin,erythropoietin,etoposide,flavopiridol,gefitinib,gln,gossypol,histone deacetylase inhibitor,ifn,il-18,interferon beta,irinotecan,kinase inhibitor,lamivudine,lithium,melphalan,mifepristone,mithramycin,mitomycin,mtor inhibitor,n-(4-hydroxyphenyl)retinamide,nsaids,paclitaxel,pd-98059,phorbol myristate acetate,phytochemical,pioglitazone,pirarubicin,protease inhibitor,protease inhibitors,protein kinase inhibitor,puromycin,resveratrol,ribavirin,selenium,sulfasalazine,tetracycline,topoisomerase-i inhibitor,topoisomerase-i inhibitors,trastuzumab,troglitazone,uvb,vitamin c,vitamin e | Arthritis, Rheumatoid; Dermatomyositis; Epstein-Barr Virus Infections; Graft vs Host Disease; Inflammatory Bowel Diseases; Liver Cirrhosis; Lupus Erythematosus, Systemic; Lymphoma, B-Cell; Lymphoma, Large B-Cell, Diffuse; Lymphoma, Non-Hodgkin; Multiple Sclerosis; Osteoarthritis; Wegener Granulomatosis |
| JAK-STAT signaling | PTPN6 | < 0.00001 | antisense oligonucleotides,epinephrine,epo,ida,il-10,il-13,il-2,il-8,interleukin-3,lif,n-acetylcysteine,sms 201-995,tgf | Arthritis, Rheumatoid; Epstein-Barr Virus Infections; HTLV-I Infections; Lupus Erythematosus, Systemic; Lymphoma, B-Cell; Lymphoma, Large B-Cell, Diffuse; Multiple Sclerosis |
| JAK-STAT signaling | JAK1 | < 0.00001 | daclizumab | Arthritis, Rheumatoid; Epstein-Barr Virus Infections; HTLV-I Infections; Liver Cirrhosis; Lupus Erythematosus, Systemic; Lymphoma, B-Cell; Lymphoma, Non-Hodgkin; Multiple Sclerosis; Pulmonary Fibrosis |
| JAK-STAT signaling | STAT5A | < 0.00001 | corticosteroids,dexamethasone,epo,prednisone | Lymphoma, B-Cell; Lymphoma, Large B-Cell, Diffuse; Lymphoma, Non-Hodgkin |
| JAK-STAT signaling | PTPN11 | < 0.00001 | - | Arthritis, Rheumatoid; Epstein-Barr Virus Infections; HTLV-I Infections; Lupus Erythematosus, Systemic; Lymphoma, B-Cell; Lymphoma, Large B-Cell, Diffuse |
| JAK-STAT signaling | TYK2 | < 0.00001 | 2c4,alpha interferon,antiviral,atra,cimetidine,cytokines,ethanol,g-csf,h2o2,ifn,il-10,il-12,il-13,il-18,il-3,il-4,il-6,il-9,insulin,interferon beta,interleukin-10,interleukin-12,interleukin-17,lif,phenylephrine,pma,tgfbeta1,tnf-alpha,tyrphostin a1,urokinase,vegf | Arthritis, Juvenile Rheumatoid; Arthritis, Rheumatoid; Liver Cirrhosis; Lupus Erythematosus, Systemic; Lymphoma, B-Cell; Lymphoma, Large B-Cell, Diffuse |
| JAK-STAT signaling | STAT5B | < 0.00001 | brl49653,dexamethasone,epo,h2o2,imatinib,progestin,retinoic acid | Epstein-Barr Virus Infections; Lymphoma, B-Cell; Lymphoma, Large B-Cell, Diffuse |
| JAK-STAT signaling | EPOR | < 0.00001 | cytokines | - |
| JAK-STAT signaling | JAK3 | < 0.00001 | leflunomide,staurosporine | Arthritis, Rheumatoid; Graft vs Host Disease; HTLV-I Infections; Inflammatory Bowel Diseases; Lymphoma, B-Cell; Lymphoma, Large B-Cell, Diffuse |
| JAK-STAT signaling | SOCS1 | < 0.00001 | 5-fu,dna vaccine,ethanol,g207,g-csf,il-5,immunosuppressive,ras inhibitor,tnf-alpha | Arthritis, Rheumatoid; Graft vs Host Disease; HTLV-I Infections; Liver Cirrhosis; Lymphoma, B-Cell; Lymphoma, Large B-Cell, Diffuse; Lymphoma, Non-Hodgkin; Multiple Sclerosis; Pulmonary Fibrosis |
| JAK-STAT signaling | GHR | < 0.00001 | il-2,insulin | Liver Cirrhosis; Lymphoma, Non-Hodgkin; Multiple Sclerosis |
| JAK-STAT signaling | STAT6 | < 0.00001 | aspirin,atorvastatin,cd40l,chondroitin,corticosteroids,csa,cyclosporin a,genistein,glucocorticoid,hgf,ifn,il-12,il-13,il-16,il-4,il-5,il-6,interferon gamma,interleukins,ionomycin,ketoconazole,leflunomide,mcp-1,m-csf,methotrexate,morphine,nac,opioids,pd-98059,phytochemical,pma,progesterone,protease inhibitor,protein kinase inhibitor,staurosporine,tumor necrosis factor,tyrosine kinase inhibitor,tyrphostin a25,uridine,vegf | Arthritis, Rheumatoid; Epstein-Barr Virus Infections; Graft vs Host Disease; HTLV-I Infections; Inflammatory Bowel Diseases; Lymphoma, B-Cell; Lymphoma, Large B-Cell, Diffuse; Lymphoma, Non-Hodgkin; Multiple Sclerosis; Osteoarthritis; Pulmonary Fibrosis |
| JAK-STAT signaling | STAT1 | 0.00001 | - | Arthritis, Reactive; Arthritis, Rheumatoid; Dermatomyositis; Epstein-Barr Virus Infections; HTLV-I Infections; Inflammatory Bowel Diseases; Liver Cirrhosis; Lupus Erythematosus, Systemic; Lymphoma, B-Cell; Lymphoma, Large B-Cell, Diffuse; Multiple Sclerosis; Osteoarthritis; Polymyositis; Pulmonary Fibrosis |
| JAK-STAT signaling | STAT3 | 0.00003 | as101,doxorubicin,flt3l,rapamycin,rituximab | Arthritis, Rheumatoid; Epstein-Barr Virus Infections; HTLV-I Infections; Inflammatory Bowel Diseases; Liver Cirrhosis; Lupus Erythematosus, Systemic; Lymphoma, B-Cell; Lymphoma, Large B-Cell, Diffuse; Lymphoma, Non-Hodgkin; Multiple Sclerosis; Pulmonary Fibrosis |
| JAK-STAT signaling | JAK2 | 0.00005 | antisense oligonucleotides,cisplatin | Arthritis, Rheumatoid; Graft vs Host Disease; Lupus Erythematosus, Systemic; Lymphoma, B-Cell; Lymphoma, Large B-Cell, Diffuse; Lymphoma, Non-Hodgkin |
| Matrix remodeling | THBS1 | 0.00027 | anti-vegf,sulindac sulfide | Arthritis, Rheumatoid; Lymphoma, Non-Hodgkin; Scleroderma, Systemic |
| NK cell mediated cytotoxicity | FYN | < 0.00001 | 12-o-tetradecanoylphorbol-13-acetate,anti-cd3 antibody,antisense oligonucleotides,antiserum,autologous tumor cells,bdnf,colchicine,cyclosporin a,dexamethasone,donepezil,ethanol,fas ligand,gdnf,geldanamycin,genistein,gp-120,herbimycin a,hgf,ifn,il-11,il-2,il-2 gene,il-3,il-4,il-6,inositol,insulin,interleukin-11,ionomycin,kinase inhibitor,leflunomide,m-csf,nsaid,nsaids,pma,quercetin,staurosporine,thrombin,tnf-alpha,tpa,transforming growth factor,tyrosine kinase inhibitors,uvb,vegf | Arthritis, Rheumatoid; Epstein-Barr Virus Infections; HTLV-I Infections; Liver Cirrhosis; Lupus Erythematosus, Systemic; Lymphoma, B-Cell; Pulmonary Fibrosis |
| NK cell mediated cytotoxicity | CD247 | < 0.00001 | fas ligand,hydralazine | Arthritis, Reactive; Arthritis, Rheumatoid; Lupus Erythematosus, Systemic; Osteoarthritis |
| NK cell mediated cytotoxicity | SH3BP2 | < 0.00001 | - | - |
| NK cell mediated cytotoxicity | VAV2 | < 0.00001 | interleukin-2 gene | - |
| NK cell mediated cytotoxicity | RAC1 | < 0.00001 | - | Arthritis, Rheumatoid; HTLV-I Infections; Inflammatory Bowel Diseases; Lupus Erythematosus, Systemic; Multiple Sclerosis |
| NK cell mediated cytotoxicity | PTPN11 | < 0.00001 | - | Arthritis, Rheumatoid; Epstein-Barr Virus Infections; HTLV-I Infections; Lupus Erythematosus, Systemic; Lymphoma, B-Cell; Lymphoma, Large B-Cell, Diffuse |
| NK cell mediated cytotoxicity | PLCG1 | < 0.00001 | 5-fluorouracil,h2o2,hsp70,il-6 | Lupus Erythematosus, Systemic; Lymphoma, Large B-Cell, Diffuse; Lymphoma, Non-Hodgkin; Multiple Sclerosis |
