# Supplementary material for: A Systems Approach to Rheumatoid Arthritis
Source: PLoS One. 2012 Dec 11;7(12):e51508. doi: 10.1371/journal.pone.0051508 (PMC3519858; doi:10.1371/journal.pone.0051508)
Supplement: Table S2 — 19 key transcription factors categorized by their associated modules in RA-perturbed network. (DOC) [file pone.0051508.s005.doc]

**Table S2**. 19 key transcription factors categorized by their associated modules in RA-perturbed network

| **Associated Modules** | **Symbol** | **P Value** | **Chemical** | **Associated Diseases** |
| --- | --- | --- | --- | --- |
| Angiogenic factors | NFAT5 | < 0.00001 | - | - |
| Angiogenic factors | ETS2 | 0.00377 | 12-o-tetradecanoylphorbol-13-acetate,bisphosphonates,csf,dexamethasone,gnrh,human chorionic gonadotropin,hydrogen peroxide,ifn,insulin,kinase inhibitor,pd-98059,pma,pthrp,tamoxifen,tpa,urokinase plasminogen activator,urokinase-type plasminogen activator | Arthritis, Rheumatoid; Osteoarthritis |
| B cell activation | POU2F1 | 0.00028 | glucocorticoid,gnrh,tas-103 | Arthritis, Rheumatoid; Epstein-Barr Virus Infections; Inflammatory Bowel Diseases; Lymphoma, B-Cell; Lymphoma, Large B-Cell, Diffuse |
| Cell cycle | ATF1 | < 0.00001 | pd 98059 | Multiple Sclerosis |
| Cell cycle | NFAT5 | < 0.00001 | - | - |
| Cell cycle | E2F3 | 0.00001 | vasopressin | - |
| Cell cycle | MYB | 0.0001 | adjuvant,antineoplastic drugs,carotenoids,c-myb antisense,cortisol,cyclosporin a,cytokines,glucocorticoid,gm-csf,h2o2,hydroquinone,il-2 gene,il-3,il-7,neomycin,nerve growth factor,pentoxifylline,protein synthesis inhibitor,red blood cells,sodium butyrate,sti571,thalidomide | Lymphoma, B-Cell |
| Cell death and survival | NFKB1 | < 0.00001 | isoflavone,pentoxifylline | Arthritis, Rheumatoid; Inflammatory Bowel Diseases; Liver Cirrhosis; Lupus Erythematosus, Systemic; Lymphoma, B-Cell; Lymphoma, Large B-Cell, Diffuse; Lymphoma, Non-Hodgkin; Multiple Sclerosis; Osteoarthritis |
| Cell death and survival | RELA | < 0.00001 | - | Arthritis, Rheumatoid; HTLV-I Infections; Inflammatory Bowel Diseases; Liver Cirrhosis; Multiple Sclerosis; Osteoarthritis |
| Cell death and survival | NFAT5 | < 0.00001 | - | - |
| Cell death and survival | TP53 | < 0.00001 | 2-methoxyestradiol,5-fc,5-fu,8-methoxypsoralen,acridine,acth,adjuvant,aminoglycosides,amprenavir,antiangiogenic,anti-vegf,atazanavir,azidothymidine,baf,bbr 3464,bcg,bec2,beta-interferon,bms-247550,c225,capsaicin,caspase inhibitor,cd40l,chemotherapeutic agent,cifn,cisplatin,c-kit ligand,clotrimazole,consensus interferon,ddathf,ddtc,denspm,depsipeptide,dht,dibenzo[a,l]pyrene,dna methyltransferase inhibitor,dna vaccine,doxorubicin,emodin,erythropoietin,etoposide,flavopiridol,fluorouracil,folic acid,fulvestrant,gene therapy agent,heparan sulfate,heregulin,hsp70,hydroxyurea,il-8,impdh inhibitors,indinavir,indomethacin,interferon gamma,ionomycin,isotretinoin,ketoconazole,leucovorin,lopinavir,mage-3,mapk inhibitors,mbo,methylprednisolone,mithramycin,mitomycin c,mitoxantrone,mixed-backbone oligonucleotide,mtor inhibitor,mycophenolate mofetil,naloxone,nelfinavir,octreotide,ol(1)p53,oltipraz,pd-98059,penicillamine,pentostatin,phenylephrine,phytochemicals,polyphenol,procarbazine,protease inhibitors,protein synthesis inhibitor,puromycin,radicicol,rapamycin,r-flurbiprofen,ritonavir,s-1,saha,saquinavir,selenomethionine,sennosides,silymarin,sodium salicylate,steel factor,tacrolimus,taxol,tetrandrine,tirapazamine,topoisomerase-ii inhibitor,tumor necrosis factor,ucn-01,udp-4,urea,ursodeoxycholic acid,uvb,vascular endothelial growth factor,vegf,vitamin c,vitamin e,xk469,yondelis,zd0473,zebularine,zinc chloride | Arthritis, Rheumatoid; Epstein-Barr Virus Infections; HTLV-I Infections; Inflammatory Bowel Diseases; Liver Cirrhosis; Lupus Erythematosus, Systemic; Lymphoma, B-Cell; Lymphoma, Large B-Cell, Diffuse; Lymphoma, Non-Hodgkin; Multiple Sclerosis; Osteoarthritis; Pulmonary Fibrosis; Wegener Granulomatosis |
| Chemokines | NFKB1 | < 0.00001 | isoflavone,pentoxifylline | Arthritis, Rheumatoid; Inflammatory Bowel Diseases; Liver Cirrhosis; Lupus Erythematosus, Systemic; Lymphoma, B-Cell; Lymphoma, Large B-Cell, Diffuse; Lymphoma, Non-Hodgkin; Multiple Sclerosis; Osteoarthritis |
| Chemokines | NFKB2 | < 0.00001 | lymphokines | Lymphoma, B-Cell; Lymphoma, Large B-Cell, Diffuse; Lymphoma, Non-Hodgkin |
| Chemokines | RELA | < 0.00001 | - | Arthritis, Rheumatoid; HTLV-I Infections; Inflammatory Bowel Diseases; Liver Cirrhosis; Multiple Sclerosis; Osteoarthritis |
| Chemokines | RELB | < 0.00001 | cd40l,n-acetyl cysteine | Arthritis, Rheumatoid; Epstein-Barr Virus Infections; Graft vs Host Disease; Liver Cirrhosis; Lymphoma, B-Cell; Lymphoma, Non-Hodgkin |
| Chemokines | REL | 0.00002 | - | Lymphoma, B-Cell; Lymphoma, Large B-Cell, Diffuse; Lymphoma, Non-Hodgkin |
| Chemokines | ETS2 | 0.00377 | 12-o-tetradecanoylphorbol-13-acetate,bisphosphonates,csf,dexamethasone,gnrh,human chorionic gonadotropin,hydrogen peroxide,ifn,insulin,kinase inhibitor,pd-98059,pma,pthrp,tamoxifen,tpa,urokinase plasminogen activator,urokinase-type plasminogen activator | Arthritis, Rheumatoid; Osteoarthritis |
| ECM organization | JUN | 0.00001 | etoposide,fr901228,ndga,pma | Arthritis, Rheumatoid; Arthritis, Reactive; Epstein-Barr Virus Infections; Lupus Erythematosus, Systemic; Lymphoma, B-Cell; Lymphoma, Large B-Cell, Diffuse; Lymphoma, Non-Hodgkin |
| JAK-STAT signaling | STAT1 | < 0.00001 | - | Arthritis, Reactive; Arthritis, Rheumatoid; Dermatomyositis; Epstein-Barr Virus Infections; HTLV-I Infections; Inflammatory Bowel Diseases; Liver Cirrhosis; Lupus Erythematosus, Systemic; Lymphoma, B-Cell; Lymphoma, Large B-Cell, Diffuse; Multiple Sclerosis; Osteoarthritis; Polymyositis; Pulmonary Fibrosis |
| JAK-STAT signaling | STAT3 | 0.00623 | as101,doxorubicin,flt3l,rapamycin,rituximab | Arthritis, Rheumatoid; Epstein-Barr Virus Infections; HTLV-I Infections; Inflammatory Bowel Diseases; Liver Cirrhosis; Lupus Erythematosus, Systemic; Lymphoma, B-Cell; Lymphoma, Large B-Cell, Diffuse; Lymphoma, Non-Hodgkin; Multiple Sclerosis; Pulmonary Fibrosis |
| Matrix remodeling | FOS | < 0.00001 | albumin,alprazolam,bisbenzimidazole,complete freund's adjuvant,desferrioxamine,doxifluridine,genistein,hmg-coa reductase inhibitor,ifn,il-3,intercalator,interferon gamma,phenylephrine,piceatannol,troglitazone | Arthritis, Rheumatoid; Lymphoma, B-Cell; Pulmonary Fibrosis |
| Matrix remodeling | NFKB1 | < 0.00001 | isoflavone,pentoxifylline | Arthritis, Rheumatoid; Inflammatory Bowel Diseases; Liver Cirrhosis; Lupus Erythematosus, Systemic; Lymphoma, B-Cell; Lymphoma, Large B-Cell, Diffuse; Lymphoma, Non-Hodgkin; Multiple Sclerosis; Osteoarthritis |
| Matrix remodeling | NFKB2 | < 0.00001 | lymphokines | Lymphoma, B-Cell; Lymphoma, Large B-Cell, Diffuse; Lymphoma, Non-Hodgkin |
| Matrix remodeling | RELA | < 0.00001 | - | Arthritis, Rheumatoid; HTLV-I Infections; Inflammatory Bowel Diseases; Liver Cirrhosis; Multiple Sclerosis; Osteoarthritis |
| Matrix remodeling | RELB | < 0.00001 | cd40l,n-acetyl cysteine | Arthritis, Rheumatoid; Epstein-Barr Virus Infections; Graft vs Host Disease; Liver Cirrhosis; Lymphoma, B-Cell; Lymphoma, Non-Hodgkin |
| Matrix remodeling | JUN | 0.00001 | etoposide,fr901228,ndga,pma | Arthritis, Rheumatoid; Arthritis, Reactive; Epstein-Barr Virus Infections; Lupus Erythematosus, Systemic; Lymphoma, B-Cell; Lymphoma, Large B-Cell, Diffuse; Lymphoma, Non-Hodgkin |
| Matrix remodeling | REL | 0.00002 | - | Lymphoma, B-Cell; Lymphoma, Large B-Cell, Diffuse; Lymphoma, Non-Hodgkin |
| Matrix remodeling | ETV4 | 0.00555 | - | - |
| NK cell mediated cytotoxicity | NFKB1 | < 0.00001 | isoflavone,pentoxifylline | Arthritis, Rheumatoid; Inflammatory Bowel Diseases; Liver Cirrhosis; Lupus Erythematosus, Systemic; Lymphoma, B-Cell; Lymphoma, Large B-Cell, Diffuse; Lymphoma, Non-Hodgkin; Multiple Sclerosis; Osteoarthritis |
| NK cell mediated cytotoxicity | NFKB2 | < 0.00001 | lymphokines | Lymphoma, B-Cell; Lymphoma, Large B-Cell, Diffuse; Lymphoma, Non-Hodgkin |
| NK cell mediated cytotoxicity | RELA | < 0.00001 | - | Arthritis, Rheumatoid; HTLV-I Infections; Inflammatory Bowel Diseases; Liver Cirrhosis; Multiple Sclerosis; Osteoarthritis |
| NK cell mediated cytotoxicity | RELB | < 0.00001 | cd40l,n-acetyl cysteine | Arthritis, Rheumatoid; Epstein-Barr Virus Infections; Graft vs Host Disease; Liver Cirrhosis; Lymphoma, B-Cell; Lymphoma, Non-Hodgkin |
| NK cell mediated cytotoxicity | REL | 0.00002 | - | Lymphoma, B-Cell; Lymphoma, Large B-Cell, Diffuse; Lymphoma, Non-Hodgkin |
| T cell activation | FOXP3 | 0.00009 | cyclosporin,glucocorticoid,tacrolimus | Arthritis, Juvenile Rheumatoid; Arthritis, Reactive; Arthritis, Rheumatoid; Dermatomyositis; Epstein-Barr Virus Infections; Graft vs Host Disease; HTLV-I Infections; Inflammatory Bowel Diseases; Lupus Erythematosus, Systemic; Lymphoma, B-Cell; Lymphoma, Large B-Cell, Diffuse; Multiple Sclerosis; Osteoarthritis; Sarcoidosis; Scleroderma, Systemic; Wegener Granulomatosis |
